# Thoracic Electrical Impedance Tomography—The 2022 Veterinary Consensus Statement

**DOI:** 10.3389/fvets.2022.946911

**Published:** 2022-07-22

**Authors:** Olivia A. Brabant, David P. Byrne, Muriel Sacks, Fernando Moreno Martinez, Anthea L. Raisis, Joaquin B. Araos, Andreas D. Waldmann, Johannes P. Schramel, Aline Ambrosio, Giselle Hosgood, Christina Braun, Ulrike Auer, Ulrike Bleul, Nicolas Herteman, Cristy J. Secombe, Angelika Schoster, Joao Soares, Shannon Beazley, Carolina Meira, Andy Adler, Martina Mosing

**Affiliations:** ^1^School of Veterinary Medicine, Murdoch University, Perth, WA, Australia; ^2^Department of Clinical Sciences, College of Veterinary Medicine, Cornell University, Ithaca, NY, United States; ^3^Department of Anaesthesiology and Intensive Care Medicine, Rostock University Medical Centre, Rostock, Germany; ^4^Department of Anaesthesiology and Perioperative Intensive Care Medicine, University of Veterinary Medicine Vienna, Vienna, Austria; ^5^Department of Surgery, Faculty of Veterinary Medicine and Animal Science, University of São Paulo, São Paulo, Brazil; ^6^Clinic of Reproductive Medicine, Department of Farm Animals, Vetsuisse-Faculty University Zurich, Zurich, Switzerland; ^7^Clinic for Equine Internal Medicine, Equine Hospital, Vetsuisse-Faculty, University of Zurich, Zurich, Switzerland; ^8^Department of Surgical and Radiological Sciences, School of Veterinary Medicine, University of California, Davis, Davis, CA, United States; ^9^Department of Small Animal Clinical Sciences, Western College Veterinary Medicine, Saskatoon, SK, Canada; ^10^Department of Clinical Diagnostics and Services, Anaesthesiology, Vetsuisse-Faculty, University of Zurich, Zurich, Switzerland; ^11^Department of Systems and Computer Engineering, Carleton University, Ottawa, ON, Canada

**Keywords:** electrical impedance tomography, animals, consensus, chest, imaging

## Abstract

Electrical impedance tomography (EIT) is a non-invasive real-time non-ionising imaging modality that has many applications. Since the first recorded use in 1978, the technology has become more widely used especially in human adult and neonatal critical care monitoring. Recently, there has been an increase in research on thoracic EIT in veterinary medicine. Real-time imaging of the thorax allows evaluation of ventilation distribution in anesthetised and conscious animals. As the technology becomes recognised in the veterinary community there is a need to standardize approaches to data collection, analysis, interpretation and nomenclature, ensuring comparison and repeatability between researchers and studies. A group of nineteen veterinarians and two biomedical engineers experienced in veterinary EIT were consulted and contributed to the preparation of this statement. The aim of this consensus is to provide an introduction to this imaging modality, to highlight clinical relevance and to include recommendations on how to effectively use thoracic EIT in veterinary species. Based on this, the consensus statement aims to address the need for a streamlined approach to veterinary thoracic EIT and includes: an introduction to the use of EIT in veterinary species, the technical background to creation of the functional images, a consensus from all contributing authors on the practical application and use of the technology, descriptions and interpretation of current available variables including appropriate statistical analysis, nomenclature recommended for consistency and future developments in thoracic EIT. The information provided in this consensus statement may benefit researchers and clinicians working within the field of veterinary thoracic EIT. We endeavor to inform future users of the benefits of this imaging modality and provide opportunities to further explore applications of this technology with regards to perfusion imaging and pathology diagnosis.

## Introduction

Thoracic electrical impedance tomography (EIT) is a non-invasive, real-time, non-ionizing imaging technique. The first reported use of EIT for medical purposes was in 1978 by Henderson and Webster (1). This technology is used in medicine to measure brain activity and gastric emptying, and for breast cancer screening and thoracic imaging (2–6). As a functional imaging technique, it has become increasingly utilised for respiratory monitoring in humans with COVID-19 illness (7–9).

**Figure 1:**
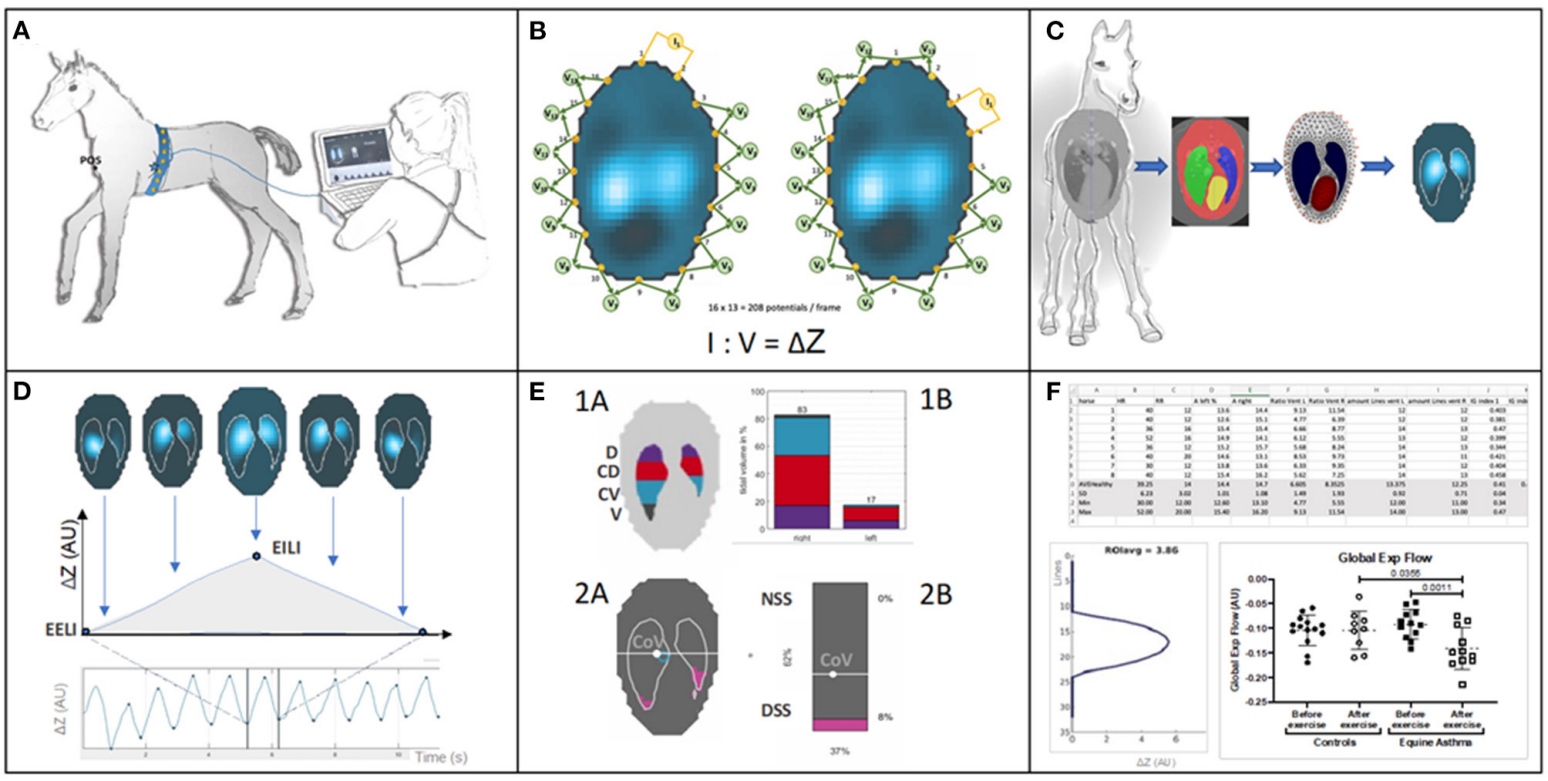
**(A)** Belt placement: Electrodes mounted on a custom-made stretchable belt are applied around the thorax of a foal. The belt is directly connected to the EIT device and from there to a stall side monitor screen, on which the generated EIT images are displayed in real-time. **(B)** Measurement principle: The EIT system applies known non-perceivable alternating current (usually between 50 and 250 kHz and an amplitude <10 mA) across a pair of “driving” electrodes and reads the voltage at the remaining electrode pairs allowing impedance calculation. The “driving” electrode pair then switches to the next set, with voltages again being measured. This continues until all pairs of electrodes have been “driven.” Based on all impedance calculations one frame is created. EIT data is constructed from these frames, usually at a rate of 50 frames per second, but this can be varied. **(C)** Application of a finite element model or mesh: To illustrate thoracic impedance changes during ventilation, images are reconstructed using a species-specific finite element model. The model is obtained from a species-specific computerised tomography image which provides anatomical information and definition of lung regions. **(D)** EIT images and waveforms: Breath related impedance change (ΔZ, y-axis) between impedance at the start of inspiration (end-expiratory lung impedance; EELI) and maximal impedance at the end of inspiration (end-inspiratory lung impedance; EILI) during respiratory cycles over time (x-axis). The image represents the impedance change between EELI and EILI. **(E)** Functional EIT images and examples of possible EIT variables: Top: 1A Thoracic image of the EIT derived variables include the 8 regions of ventilation. The eight regions are derived from anatomical sides within the lung parenchyma right (R), left (L), and anatomical region dorsal (D), centro-dorsal (CD), centro-ventral (CV), ventral (V) within each side. 1B The same variables represented in a bar graph illustrating division of ventilation within these regions. Bottom: 2A Thoracic image of additional EIT variables describing: centre of ventilation (CoV) and its vertical and horizontal orientation within the finite element model and the non-dependent (NSS) and dependent (DSS) silent spaces depicted by pink shading (taken from ibex, Sentec, Switzerland). 2B A bar chart representing the preceding variables in percent (%). **(F)** EIT measures and analysis of variables: examples of numeric output of variables and graphical illustration of results after further processing for off-line analysis.

Impedance measurements are based on application of alternating currents applied via pairs of electrodes and measurement of the resulting voltages at the remaining electrodes. From the distribution of these voltages, regional impedance changes are calculated (2, 3). By mounting the electrode pairs on an electrode-belt which is applied around the thorax of a subject, thoracic EIT can monitor the ventilation- and cardiac-related changes in regional impedance based on subsequent reconstruction of cross-sectional images of the thorax (Figure 1).

The first report of EIT applied in veterinary medicine described the distribution of ventilation in pregnant Shetland ponies (10). Since then, more than 35 papers describing applications in veterinary medicine across multiple animal species have been published (see reference list and Appendix 1).

Thus far, data collection, analysis and reporting among these papers have not been standardised, which has led to inconsistencies in interpretation and reporting. As applications of EIT are further explored in animals in both clinical and experimental settings, the need for uniform reporting has become apparent.

This consensus statement has been prepared by veterinary clinicians/researchers and biomedical engineers with experience using thoracic EIT in a variety of animal species. The aim of this paper is to lay the groundwork for thoracic EIT in veterinary medicine by explaining the technical principles of EIT to users, clarify nomenclature and provide recommendations on data collection in animals, thus providing a framework for consistent data reporting and analysis. Translational animal studies for human medicine have only been included if relevant to veterinary medicine. This document is intended as a guideline only and not recognised as a standard of practice. The recommendations are not exclusive protocols to dictate procedures. They have been based on practical clinical experience and a consensus of expert opinions. Wherever possible, evidence-based literature has been cited. However, further evidence is required to support some of these recommendations.

## Technical Background on EIT

### General

Different tissues have an individual innate resistance to electrical current. When alternating current is used, this resistance is referred to as impedance. Air is a poor conductor of electrical current while fluid (e.g., saline) is a good electrical conductor. Changes in air volume during respiration correspond to changes in conductance; more air during inspiration will correspond to higher electrical impedance, while less air during expiration corresponds to lower electrical impedance. The EIT system applies known current across a pair of *driving* electrodes and reads the resulting voltage at the remaining electrode pairs. After measuring the voltage, the *driving* electrode pair then switches to the next set, with voltage again being measured and impedance calculated. This continues until all pairs of electrodes have been *driven*. Most single frequency systems apply an alternating current between 50 and 250 kHz at an amplitude of 3–10 mA (3). The current applied is typically imperceptible in people, and no known adverse effects have been reported in its use, even in critically-ill human neonates (11).

A single EIT frame is produced once a *driving* current has been applied to every electrode pair and resultant impedance changes have been calculated. One EIT frame generates one reconstructed image usually with a 32 × 32 pixel matrix at a given time point during a breath. Most EIT systems achieve approximately 50 frames per second (3). In comparison to conventional thoracic imaging technologies (e.g., computed tomography and magnetic resonance imaging), EIT creates an image of relatively low spatial, but high temporal resolution. Analysis of multiple sequential raw images leads to the generation of global, regional and pixel-level impedance waveforms (3).

### EIT Systems

At the time of writing, the following EIT systems are commercially available:

1) BB^2^/BBVet (Sentec, Landquart, Switzerland) (32 electrodes)
2) Enlight 2100 (Timpel, Eindhoven, The Netherlands) (32 electrodes)
3) Enlight 1800 (Timpel/Dixtal, Eindhoven, The Netherlands) (8 electrodes)
4) GOE-MF II (Carefusion, Höchberg, Germany) (16 electrodes)
5) Pioneer (Sentec, Landquart, Switzerland) (32 electrodes)
6) PulmoVista® 500 (Dräger Medical, Lübeck, Germany) (16 electrodes)
7) Sheffield MK 3.5 (Maltron Inc, Rayleigh, United Kingdom) (8 electrodes)
8) Sigma tome II (École Polytechnique, Montréal, Canada) (16 electrodes).

Most research on animals to date has used the Sentec systems, although use of all except the Sheffield MK 3.5 (Maltron Inc, Rayleigh, United Kingdom) has been described (Appendix 2). Other systems have been described in translational research (12–14).

Most commercially available systems use 32 electrodes placed circumferentially around the thorax, mounted equidistantly on a stretchable belt; however, some systems use 8 or 16 electrodes (2). A greater number of electrodes improves spatial resolution, as well as providing some degree of redundancy if one or more electrodes fail or have poor contact (2). Redundancy is most easily observed in systems with only 8 and 16 electrodes, where the loss of electrical contact or function of one electrode can lead to complete loss of data. In 32 electrode systems, such as Sentec (Sentec, Switzerland), data collection is still possible with a loss of up to six electrodes, but data quality will be reduced (15).

There are several patterns of *driving* electrodes. These include adjacent, skip (e.g., skip-4 means that electrodes 1 and 6 are *driven* together) and square. An example of *adjacent-drive* is given in Figure 1B. These *drive* patterns are typically set by the manufacturer of each EIT system, although some skip-patterns are adjustable. Examples of patterns used by available systems include skip-4 (Sentec system), adjacent (Dräger) and skip-3 (Timpel) patterns.

### Single-Plane and Two-Plane EIT

EIT measures electrical impedance over a three-dimensional (3-D) volume, extending cranially and caudally from the belt to approximately half of the thoracic diameter, to produce a lens shaped area of interrogation (termed vertical sensitivity) (3, 16). Thus, single-plane EIT detects impedance change in thoracic structures cranially and caudally to the belt position, particularly in the centre of the thorax. This lens-shaped area of thorax is subsequently displayed in a two-dimensional (2-D) image.

**Figure 2:**
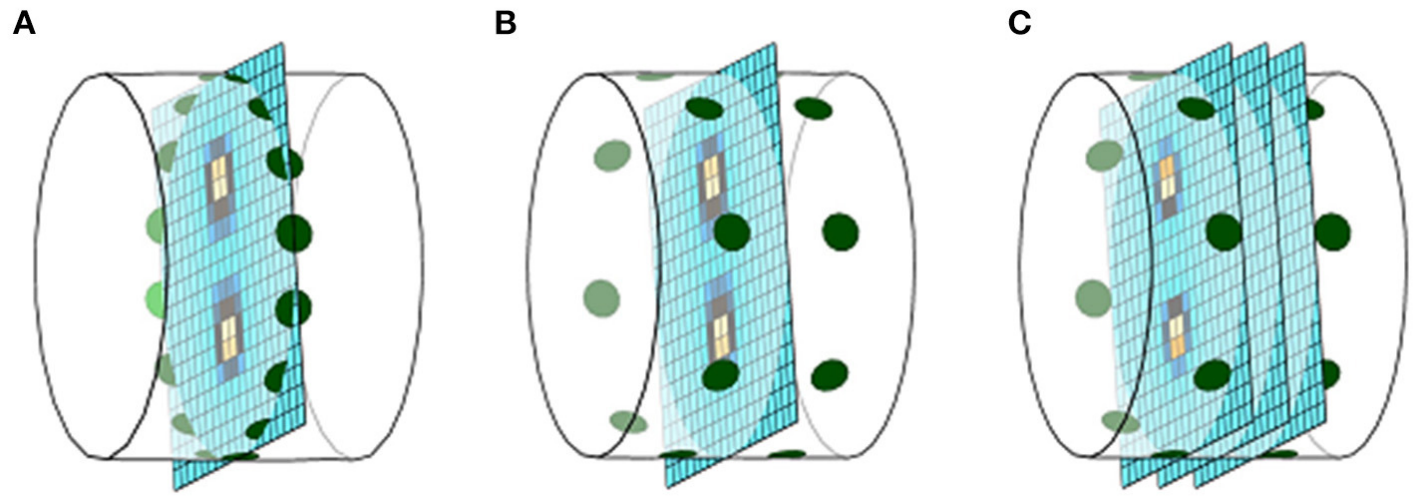
Illustration of EIT reconstruction in 2D and 3D (left to right): Cranial is towards the left of the image and caudal to the right. **(A)** A single electrode and image plane. **(B)** A single image plane with multiple electrode planes. **(C)** Multiple electrode and image planes.

**Figure 3:**
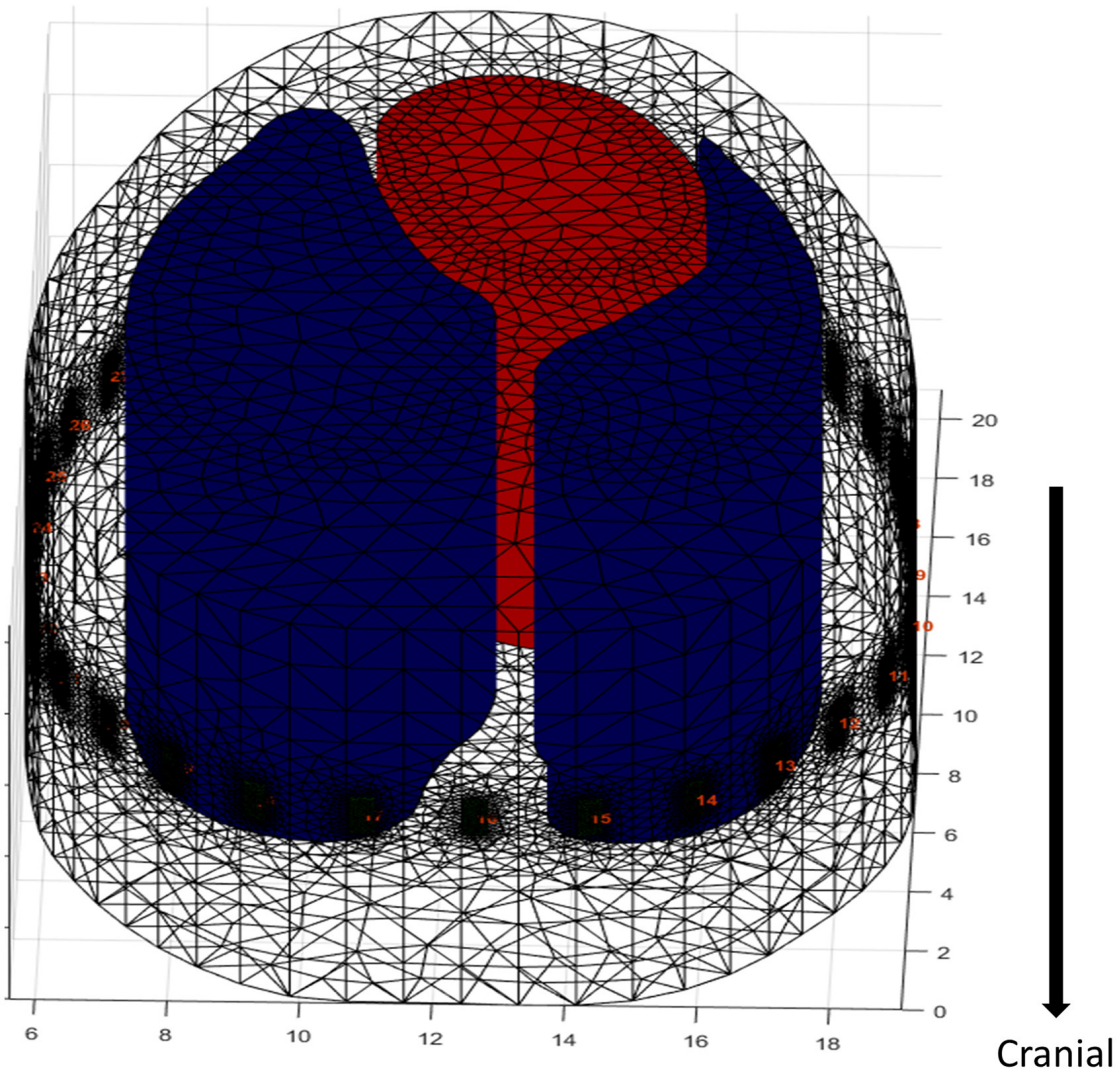
Extrapolation of an FE model in a dog. The electrodes are artificially placed into the model around the contour at the level of the 5–6th intercostal space. The cranial and caudal direction does not account for anatomic changes in ROI contour, as it is taken from a single CT slice resulting in no variation in the diameter of the lung model. A 2-D FE model would miss data capture as EIT does not measure a single plane but captures data in an ellipse reducing in sensitivity as distance from the electrode plane increases, hence the need for increased depth of the FE model. However, with the lack of variation in diameter there is potential of artefactual data (black dots = electrodes, red = heart, blue = lungs).

Two-plane EIT has recently been developed and evaluated in a horse (17). Two-plane EIT detects impedance change over a greater craniocaudal volume of tissue, providing insight into complex ventilation and perfusion heterogeneity that might occur in a longitudinal direction. Two-plane EIT is displayed as multiple transverse slices through the longitudinal axis and can be reconstructed into a 3-D image (Figures 2, 3). This is still in development and not yet adopted into practice (17).

Recommendation: EIT systems and image reconstruction techniques should be described in manuscripts to include:1) EIT system (manufacturer details, following journal guidelines), 2) number of electrodes, 3) pattern of *driven* electrodes using pair *drive* technique, 4) frames/second, 5) electrode position (plane).

### Finite Element Models and Functional Regions of Interest for EIT Image Reconstruction

#### Finite Element Models or “Meshes”

Initially, images were reconstructed on circular planes that had no anatomic detail. However, this method did not allow for precise mapping of information to anatomical areas and was primarily used to look at changes over time. In order to assess the location of impedance change within the thorax, the software requires a model of thoracic anatomy on which to map these impedance changes. As the majority of changes of impedance in the thorax relate to movement of air and liquid (blood) within the lungs, heart and vasculature, these organs are predefined as regions of interest (ROIs). The areas outside the ROIs typically have no important change in impedance from breath to breath (or beat to beat) and as they are not of clinical interest and they are usually filtered out (Figure 1C).

To enable the EIT software to reconstruct the impedance curves onto the ROI and to allow evaluation of disease from a single examination, construction of a finite element (FE) model, or “mesh,” is required. In addition to improving image accuracy a FE model allows the EIT software to recognise electrode position, which is particularly important in the case of poor electrode contact or failing electrodes (13).

Finite element model creation is based on cross-sections of the thorax at the position of the belt to map where the outer and inner contours of the lung and the heart are within the thorax (Figure 1C). The slice is typically chosen to minimise any inclusion of the heart and abdominal viscera and maximise the available lung to thorax ratio (18). Cross-sectional advanced imaging (i.e., CT) (10, 19) or post-mortem specimens (frozen or fresh) are used (20, 21) to provide anatomic detail. Note that for fresh samples recumbency may change the shape and position of organs due to gravitational effects which should be taken into account. CT images are typically collected during an inspiratory pause (Figure 1C). An alternate method of FE model generation uses gypsum casts of live animals at the intended belt position to provide an outer contour, with the lung ROI presumed based on anatomic features (22).

**Figure 4:**
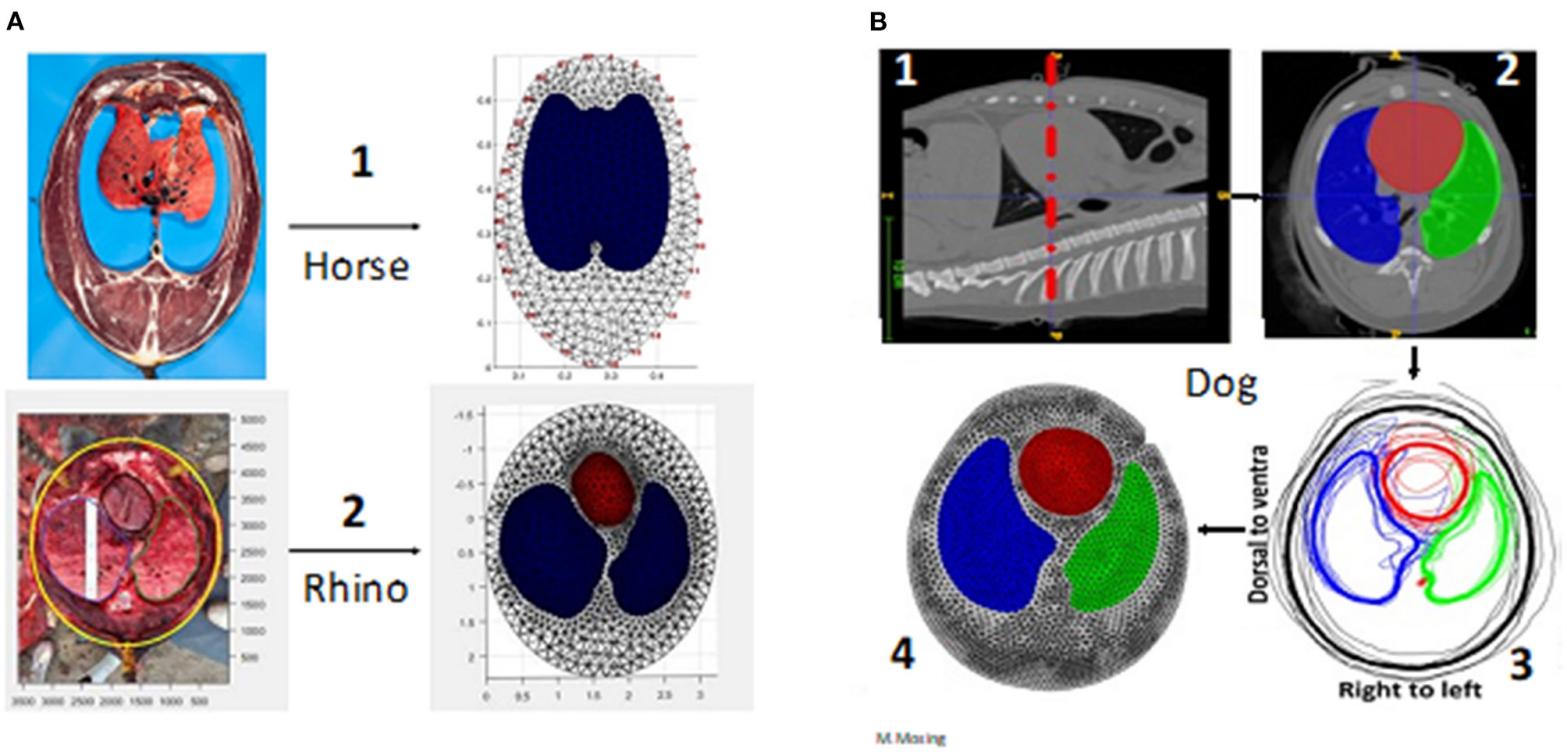
Creation of finite element models. **(A)** Via anatomical model. A1 is a frozen slice of a horse specimen, A2 a fresh dissection of a rhino thorax *in situ*. **(B)** Via computed tomography (CT) imaging in the dog (beagle). B1. CT image of the correct anatomical position (the belt position highlighted with red dashed line) B2. Segmentation of the regions of interest (the heart and lungs red = heart; green = left lung; blue = right lung) B3. Averaging from multiple animals (Thick black line = averaged outer contour) B4. The completed finite element model.

For single-plane EIT, one slice, usually corresponding to the 5th to 6th intercostal or intervertebral spaces is used to create the mesh (10, 12, 19). Manual delineation of the heart and lung ROI is performed using dedicated segmentation software. These delineations are then automatically averaged, if possible, from multiple animals, and the segments are converted into a finite element model using mathematical modelling software (e.g., Matlab, Mathworks, Natick, MA, USA). An accurate thoracic mesh improves image reconstruction and allows accurate calculation of hypo-ventilated lung areas (dependent Silent Spaces) and therefore a better display of data. Finite element model generation is summarised in Figure 4.

Meshes allow *mapping* of impedance changes onto the model of the thorax at the level of the belt, accounting for the location of the lung regions and separating them from the heart and abdominal organs (23, 24).

A major limitation of FE mesh use primarily relates to their representation of an entire species, when relying on data from low numbers of animals. Typically, FE models created using CT images have evaluated 6–8 animals (10, 12) while large animal studies based on cadavers have used 1–2 animals only (20, 21). For non-domestic animals, there may be limited numbers on which to base an FE model, although CT images of an individual animal can be used to generate FE models for subsequent EIT data analysis that will be, by definition, individualised for that animal (25).

In addition, intra-species differences should be considered. For instance, differences in chest shape and lung size that occur with age (e.g., neonate vs. adult) or breed (pony vs. Thoroughbred, barrel vs. deep-chested dogs) might require different meshes to most accurately identify impedance changes within the ROIs.

Given the methodology of FE model generation using fixed anatomical or imaging sources, their use might lead to unexpected or unintended exclusion of certain impedance data by predefining the ROI. This might cause a loss of information under certain circumstances such as with high tidal volumes where the lung area exceeds the predefined ROI.

A final limitation of FE model generation from a single slice is that the model assumes that the outline of the ROI does not change cranially or caudally, which is untrue (Figure 3). Given the vertical sensitivity of single-plane EIT (Figure 2), this may affect data interpretation in certain circumstances.

#### Functional ROI

An alternative to the application of a thoracic mesh is the determination of ROIs based on impedance change in a subject at a time-point. Functional ROIs (fROIs) are determined by excluding areas of the thorax with low impedance change (26). Recommendations for inclusion limits of fROIs are pixels within 20–35% of the maximum standard deviation of the impedance change. Below 20% there is a lot of electrical interference outside of the actual lung fields (27). However, this may exclude under-ventilated lung tissue (26). Specifically, areas of over-distension or atelectasis leading to low air movement and thus low impedance change (e.g., Silent Spaces when a species-specific mesh is used) will be incorrectly interpreted as non-lung tissue, leading to overestimation of lung health and function as these areas will no longer be counted as lung tissue when they should be, thus actual lung function will be worse than is shown by the EIT.

An example of applied fROIs is the evaluation of the *ventilated area* (see global variables) (28). This describes the area over which impedance change consistent with ventilation is observed. It does not describe the anatomical, but the functional lung area and thus fROI is suitable.

Recommendation: Species-specific finite element models should be created from the average of representative animals, using cross-sectional anatomic data. For single plane EIT, the chosen anatomical region should maximise the lung ROI within the chest, typically at the 5th to 6th intercostal space at half thoracic height. Finite element models should be used when variables corresponding to anatomic locations (e.g., lung or cardiac ROI) are described; otherwise, they may be unnecessary and functional ROIs can be used as an alternative.

## Author Experiences

### Data Collection From Consensus Members

To determine current practices used in the collection and analysis of EIT data, the veterinary literature was examined, and veterinarians and biomedical engineers involved in veterinary EIT research were contacted in early 2021. Participants received a questionnaire including open questions relating to general application and data analysis (results are given in Appendices 2–6 in Supplementary Material).

### Consensus Members

Twenty-two experts were contacted and twenty-one agreed to participate in a consensus group. A group of nineteen veterinary surgeons, of them seven veterinary specialists (diplomates of the European or American College of Anaesthesia and Analgesia (*n* = 7) or American College of Veterinary Internal Medicine (Large Animal Internal Medicine) (*n* = 4) or European College of Bovine Health Management (*n* = 1) and two biomedical engineers from Australia, Austria, Brazil, Canada, Switzerland and the USA contributed to the final guidelines.

Results from the collected information shows that the consensus group members have experience in electrical impedance tomography (EIT) in a variety of species from chickens to rhinoceros with the majority of the experience in horses.

### Animal Status

Veterinary EIT can be used in animals that are conscious, un-sedated, sedated, anaesthetised or cadaveric (where mechanical ventilation provides airflow). Animals can be positioned standing, in lateral, dorsal or sternal recumbency. EIT can be used in animals with or without endotracheal intubation or facemasks. Breathing can be spontaneous, assisted or controlled via manual or mechanical ventilation.

### Belt Design

#### Belt

A durable and stretchable fabric that can be thoroughly cleaned is recommended (29). Existing belts for animals use neoprene or other elastic fabric. Inter-electrode distances should be equal to correspond with the reconstruction model and the belt should exert uniform tension around the convex circumference.

**Figure 5:**
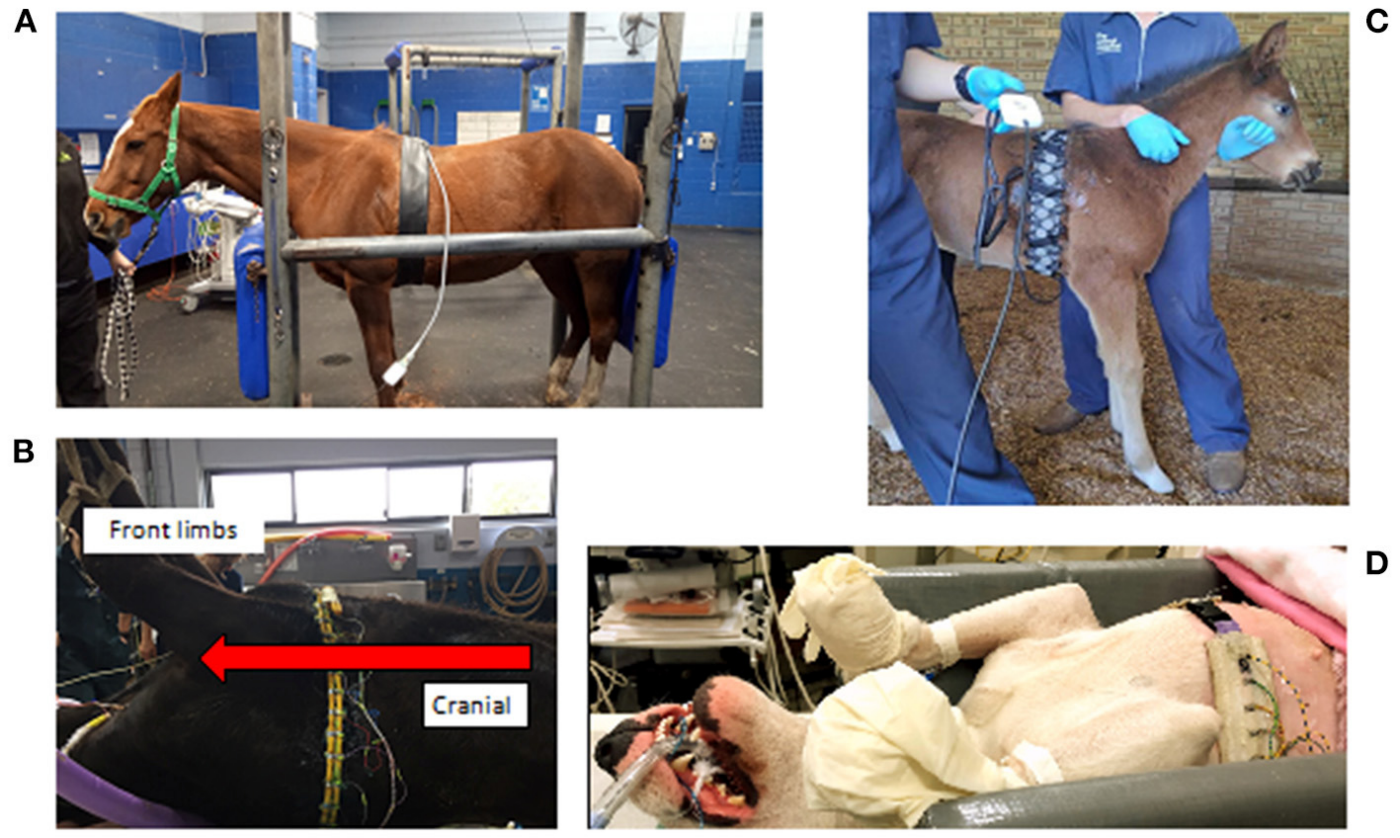
Examples of various EIT belts available. **(A)** A single-plane horse belt made of neoprene fabric of 10 cm width with integrated electrode and cable, positioned on a sedated horse in a standing position and connected to the data acquisition computer. **(B)** A single-plane cow belt made from two pieces of rubber tubing with individually wired electrodes and cables, positioned on an anaesthetised cow in dorsal recumbency with the cranial part of the cow to the left of the picture. **(C)** A single-plane foal belt made from elastic fabric of 10 cm wide with push buttons and a detachable cable belt with integrated electrodes positioned on a standing un-sedated foal. **(D)** A single-plane dog belt made from elastic fabric with single wired electrodes positioned on a dog in dorsal recumbency.

**Figure 6:**
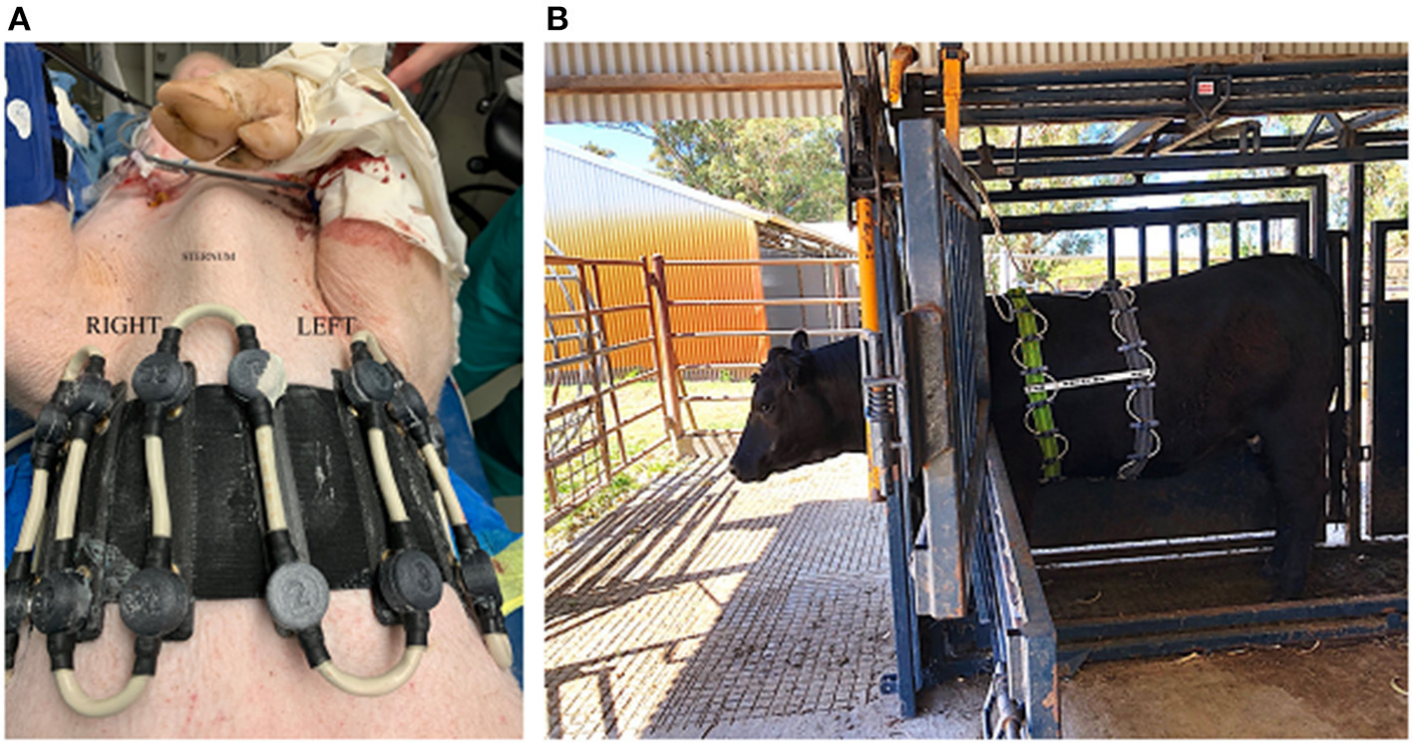
Two-plane EIT belts. **(A)** On a pig lying in dorsal recumbency (cranial is to the top of the picture) with 32 electrodes in two electrode planes on one electrode belt and one continuous cable belt. **(B)** On a standing cow restrained within a crush, consisting of two electrode belts with 16 electrodes each, with spacers between the rows, and a separate cable belt on two stretchable electrode belts. In the pig and cow EIT set-up, the separate cable belt attaches to the stretchable electrode belt via metal fastenings that can be attached and detached as necessary.

The belt can be a single-plane belt with either 16 or 32 electrodes, or a two-plane belt with two rows of 16 electrodes, creating a total of 32 electrodes (30). Two-plane belts can then be used to obtain one or two-plane data, depending on whether a single row or both rows of electrodes are used, resulting in 2-D and 3-D EIT, respectively (Figures 4–6).

Recommendation: The use of a durable and stretchable material e.g., neoprene that can be easily cleaned with an appropriate species-specific belt closure (e.g., plastic clasp) for safe use is suggested.

#### Electrode

**Figure 7:**
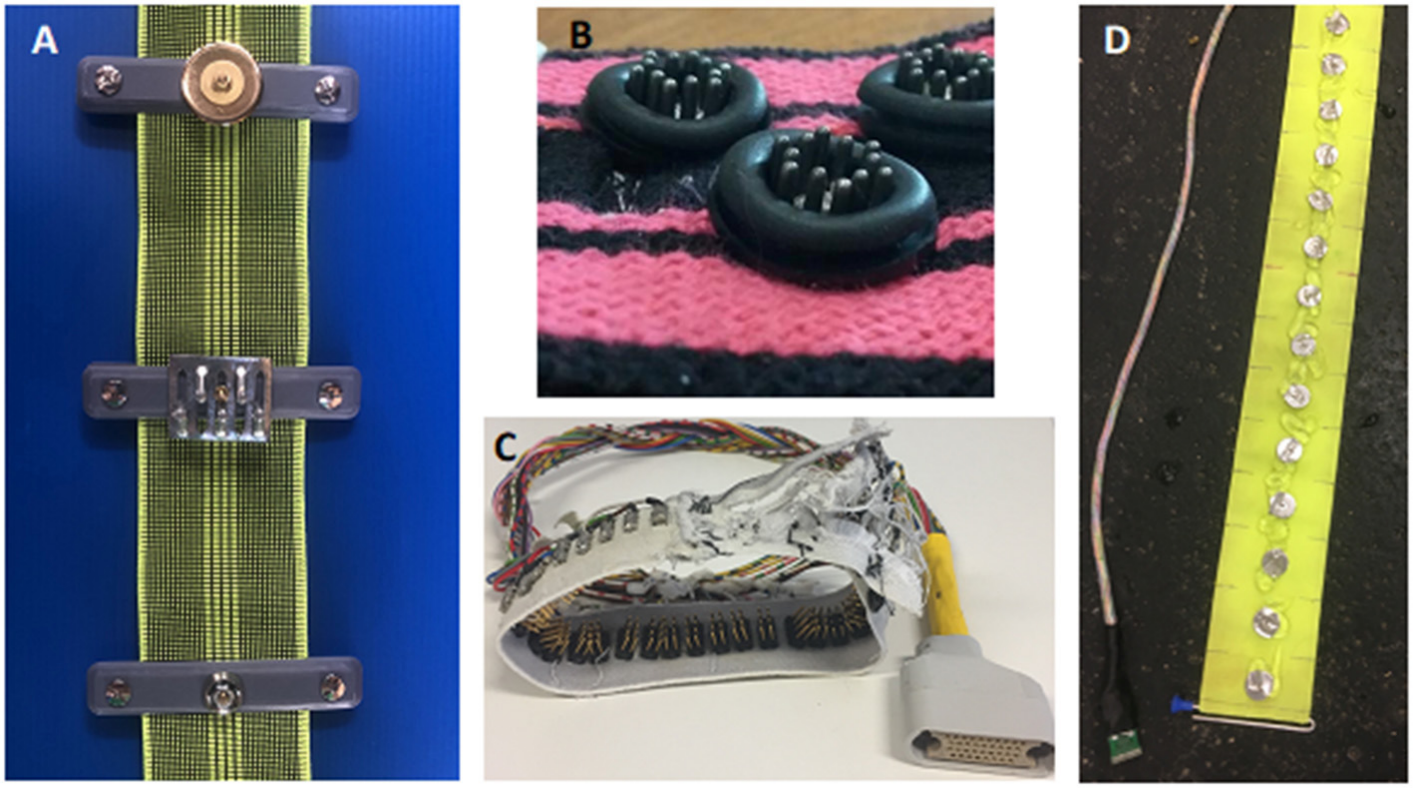
Example of electrode designs. **(A)** Three Electrode designs for a cow belt top- gold plated flat washer, middle- zinc galvanised spike, bottom- zinc plated rivet. **(B)** Electrode design for a dog belt. The electrode consists of stainless-steel spikes encapsulated by a rubber ring to allow targeted application of gel. **(C)** Electrode design for a chicken belt used in lambs and chickens. The electrode is constructed from multiple thin stainless steel spikes to allow penetration through the hair or feathers. **(D)** Electrode design for a horse belt. The electrodes are medical grade stainless steel flat washers.

Electrode design requires a material that conducts electricity, is durable and will not damage the skin of the animal. This is especially important when a recumbent animal is lying on the electrode. Materials that have been used include gold-plating, brass, medical grade steel, and galvanised and plated zinc. Evaluation of materials suggest that gold-plating reduces skin contact impedance, the resistance to current between the electrode and skin surface, when compared to zinc derivatives, in large animal EIT, leading to increased image resolution when reconstructed (31 “In Press”) (Figure 7A).

The design of electrodes includes round flat washers, pins, spikes, custom-made screws and rectangular electrodes. The design of the electrode should reflect the use of the EIT system.

For standing animals with long coats, pins may be appropriate to allow penetration through the thick hair. Washers have been shown to be suitable for recumbent and standing animals. Flat washers are suitable for animals that are recumbent as they do not cause skin damage. The diameter of the electrode and distance between electrodes should be in the magnitude of 1:3 to 1:5, depending on species and thoracic circumference to allow high enough resolution for image reconstruction (31).

Recommendation: Electrode material and design has been scientifically evaluated for cattle. The ideal electrode for other species is yet to be determined and the recommendation is to trial electrode material and design in pilot projects before conducting scientific EIT studies.

#### Skin Preparation

The skin/hair of any animal does not need to be clipped but should be wet and clean prior to application of the belt, with or without the addition of hypertonic, isotonic saline and / or low conductive ultrasound gel (Appendices 3, 4). Air does not conduct electrical current. Therefore, air pockets in the fur and between electrode and skin should be eliminated since they will increase skin contact impedance and cause artefacts during data collection.

#### Gel

**Figure 8:**
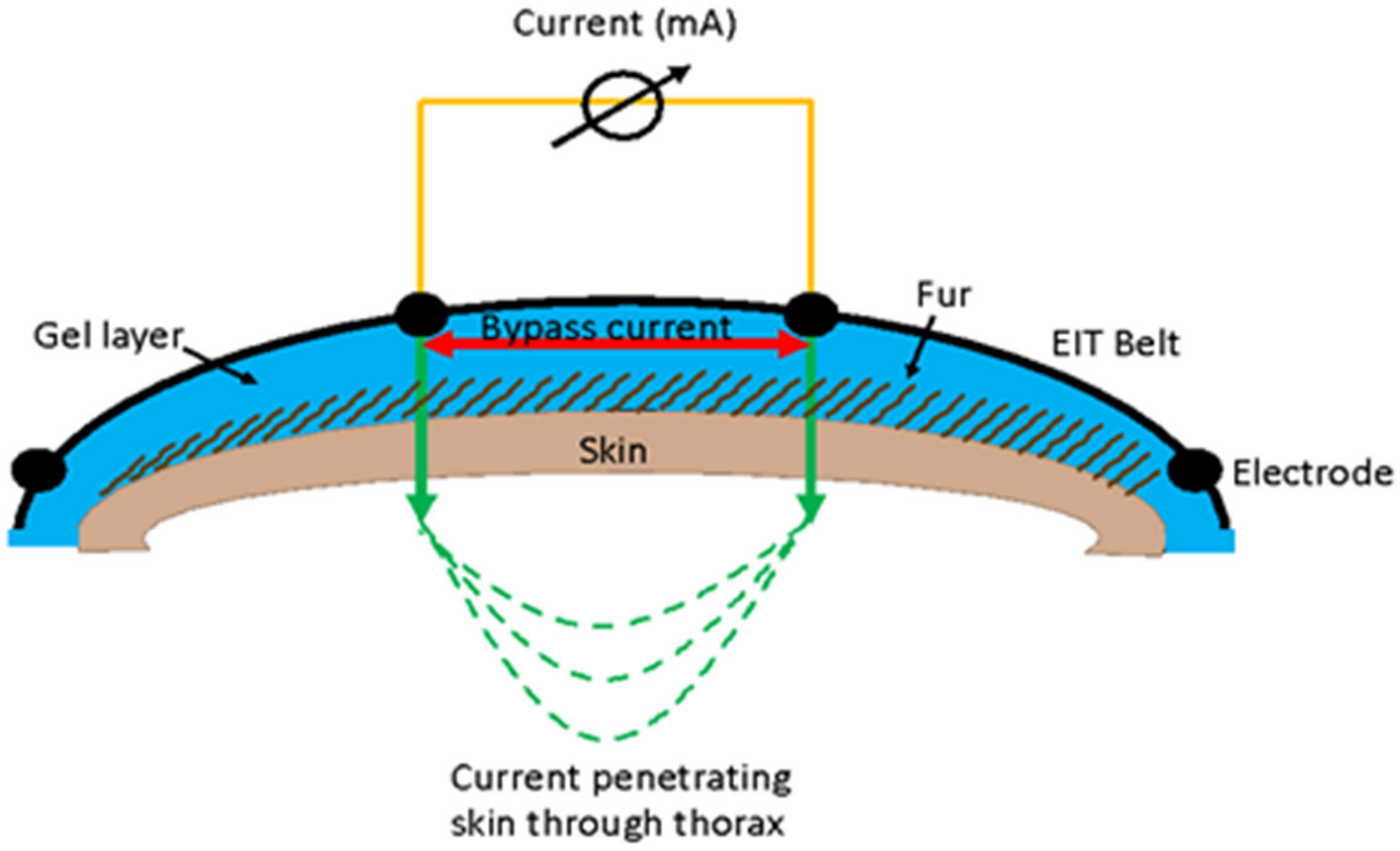
Illustration of the use of gel in EIT measurement and how a bypass current can occur between electrodes where the current flows in the gel layer (red arrow) rather than going through the skin (green arrow).

Use of conducting gels can lead to a shorting (bypass current) since the current can move through the gel layer on the skin surface rather than through the tissue and therefore thorax (Figure 8). Use of low conducting (e.g., ultrasound or obstetric) gel is advised as this is water soluble, has a thicker consistency, and acts as a coupling medium to reduce air between the electrode and the skin.

#### Belt **P**lacement

In most species the belt should be placed vertically using the landmarks for the 5–6th intercostal space (ICS) as the midpoint of the vertical height of the thorax. This location marks the region of the thorax in cows and dogs where the largest portion of the lung is detected and where there is the least interference from the heart, and gastrointestinal tract (22, 32–34). The recommendation for either the 5th or 6th ICS is due to variation in anatomy; for instance, in cattle the 5–6th ICS is recommended due to the diaphragm extending cranially to the 6th ICS (33, 35). For a few species, dogs foals and chickens there are special recommendations (Appendix 3) due to differences in anatomy. In foals, the ideal belt position is at the intersection of a horizontal line at the level of the tuber ischium through the 6th intercostal space. This was verified based on a radio dense marker belt applied during thoracic computerised tomography studies (Sacks et al., in preparation).

Small areas of clipped hair can be used as markers. Clipping can be performed after initial placement of the belt. These markers ensure repeatable positioning of the belt when an animal is moved or the belt is removed and replaced between measurements (36, 37).

Recommendation: Skin preparation should include the wetting of the hair/coat and low conducting ultrasound gel should be used if using gel. There is no need to clip the hair apart from where marks are necessary to replace the belt in the correct position. The belt should be placed vertically at the level of the 5–6th intercostal space dependant on the species.

### Data Collection

In order to acquire stable measurements, electrodes must have certain functional characteristics including low skin contact impedance, stable skin-electrode contact over time and small skin-electrode contact impedance changes during breathing. Loss of any of these characteristics can result in electrode failure. Information on electrode failure is displayed in real-time on most EIT monitors. Once these functional aspects have been met images can be generated and raw EIT data collected.

To ensure an adequate number of breaths available for analysis, it is recommended to record at least 1 min of stable breathing at each time point of interest to allow off-line analysis of a sufficient number of representative breaths (10, 34, 38–41). In conscious animals, the animal should be as still as possible to avoid movement artefacts, with the head in a straight position to avoid redirection of inhaled gas to one specific lung (22).

Recommendation: Low skin contact impedance is required for data collection and a minimum of three breaths for data reconstruction after a period of stabilisation.

### Image Reconstruction

The impedance can be calculated between each pair of electrodes from the current and measuring the voltage (Figure 1B). Most current EIT image reconstruction relies on time-difference reconstruction (tdEIT).

Time-difference EIT uses a baseline condition with which to compare impedance images. This helps eliminate or reduce instrumentation and finite element model errors (3). Disadvantages of tdEIT include that it cannot identify tissues that have no change in condition (e.g., masses) (3).

Various reconstruction algorithms have been produced which vary in their ability to accurately discern the shape, regions of interest, noise and image resolution of raw EIT images. Overall, the Graz consensus Reconstruction algorithm for EIT (GREIT) is the most commonly method used for image reconstruction in the existing veterinary literature. Gaussian spatial and low-pass temporal filters have also been used to reconstruct images (22, 42).

By using the GREIT reconstruction algorithm, the voltage measurements from a single cycle of current application, referred to as a frame, gathered at the rate of 50 frames per second can be used to create a raw image. The result of raw EIT image generation and subsequent analysis using tdEIT is a series of two-dimensional raw images that demonstrates the change in overall impedance in the ROI over time (Figure 1D). These raw images can be analysed after undergoing reconstruction through a variety of proprietary or experimental algorithms. The initial output of these algorithms is typically a waveform representing change in impedance plotted over time. Pulmonary perfusion and cardiac-related impedance changes are usually much smaller than ventilatory changes; contrast agents such as saline can be used to enhance the impedance changes and improve the ability of creating perfusion images and waveforms (2). If impedance change associated with perfusion is relatively large frequency filtering (and subsequently regional or pixel filtering) can be used to suppress these non-ventilatory signals if interference is observed. Usually, waveforms (also known as impedance curves) are produced for global (averaged) ROI impedance change. However, they can also be generated at regional, line and pixel levels.

The above predominantly applies to single-plane EIT data. However, two-plane EIT can be handled similarly, with separate waveforms created for as many *slices* as required. As described previously, there are some differences expected between 2D images created using single-plane EIT data and 2D images created using two-plane EIT data when evaluating similar slices of the thorax (Figure 2).

Time-difference EIT algorithms use a predefined point with which to compare subsequent impedance change. This reference point may be the mean of all measurements, a physiologically meaningful event such as end-inspiration or end-expiration or moving averages. The latter is typically used for real-time evaluation of EIT images. All techniques have advantages and disadvantages that should be considered prior to implementation (2).

### Data Analysis

Data analysis software available includes ibeX (Sentec, Switzerland), Matlab (Mathworks^®^, USA), Octave (GNU public license, USA) and custom software through EIDORS (GNU public license, USA) (Appendix 5).

Data can be analysed with or without lung contours; to allow correct interpretation of the data it is essential to clarify whether an FE model has been used in the analysis when reporting the results.

**Figure 9:**
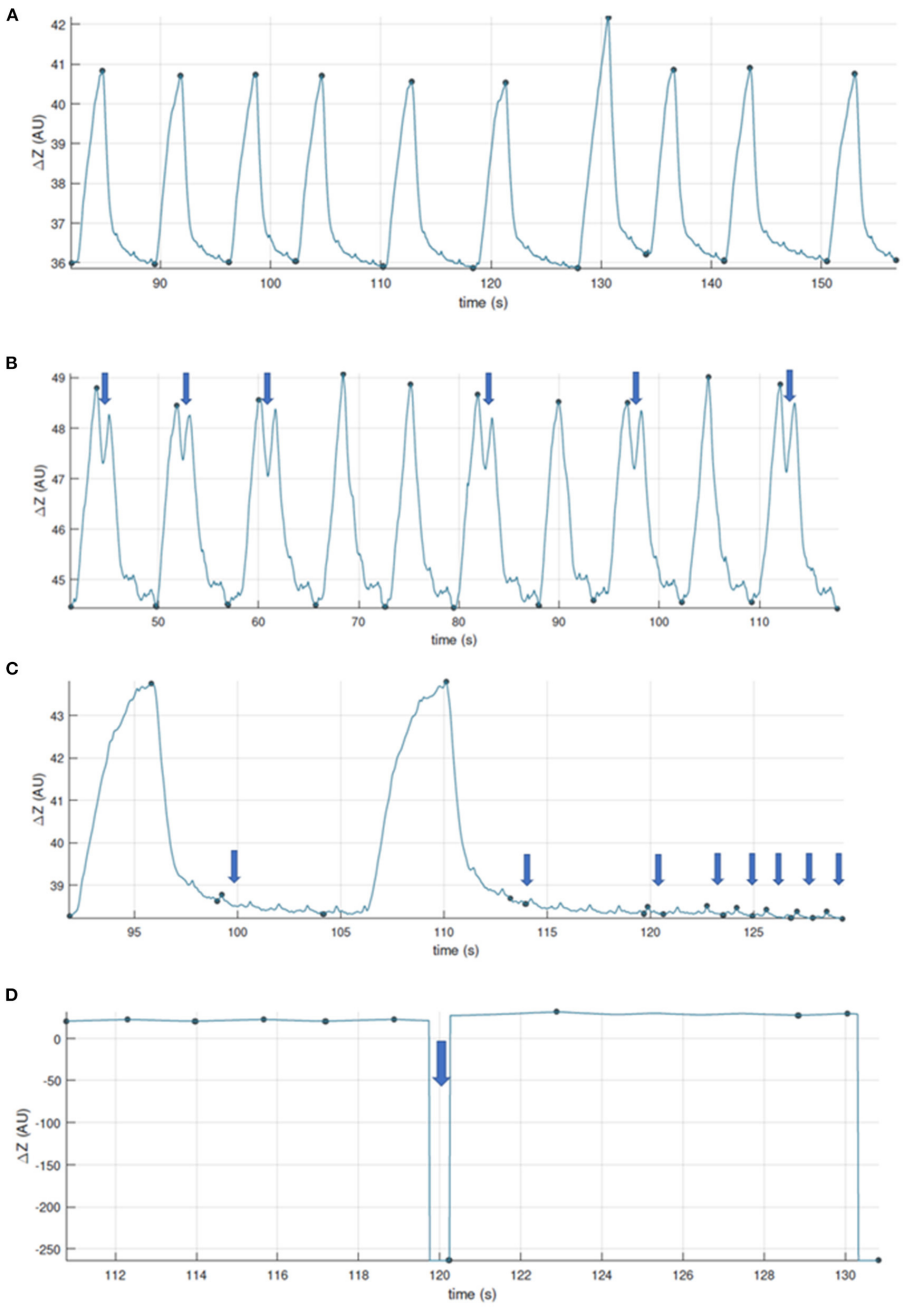
Examples to aid with inclusion and exclusion of EIT breaths for further analysis. **(A)** Good quality recording, artifact free. **(B)** Good quality recording showing some two-stage breaths. These breaths can be included if they were observed and recorded as special breaths*. Video-recording thoracic excursions during data collection can aid in discerning which baseline drifts are to be included (observed as thoracic excursion) or excluded (observed as movement of the limbs or neck). **(C)** Good quality recording with presence of cardiac-related signals. When assessing ventilation, breaths whose recognition may be affected are to be excluded, or the data further filtered. **(D)** Recording whose quality is affected by calibration or electrode fail artifacts. *Specific analysis may be required for special breaths depending on factors such as software used. Thoughtful EIT data treatment has to be especially considered for special breaths when included.

For analysis, the number of breaths analysed for one measurement time point ranges from a minimum of three breaths up to 22 breaths (28, 31, 32, 39, 43). Most commonly, 10 breaths are analysed as a representative number of breaths for a measurement period. As a general guide for breath selection, consistent, representative, uniform, artefact-free breaths should be used (Figure 9). While consecutive breaths are ideal, non-consecutive breaths can be used for analysis.

Once the breaths have been selected, a variety of variables can be calculated and exported as videos, numerical values, or a series of individual breath images. The data acquired can then be analysed on a global, regional, line or pixel scale.

Recommendation: We recommend the GREIT reconstruction algorithms for most image reconstruction. Data analysis can be performed using numerous software programs and selection should be stated when constructing manuscripts.

### Statistics

The value of EIT assessment of thoracic physiology goes well-beyond traditional modalities of imaging, being able to reconstruct a dynamic, 3-D image of lung ventilation and perfusion, as well as changes in cardiac function. Early work and analysis of data has focused on validating the instrumentation but in fact, that has been difficult since there is no standard with which it can be compared. The earliest work simply validated it for estimation of tidal volume, mainly to align veterinary clinicians into accepting its application as a monitor of respiratory function. Its comparison to spirometry was presented since spirometric tidal volume estimation is accessible, and well-established, despite its flaws (39). This early work demonstrated that impedance does follow a linear relationship with spirometric tidal volume but closer examination shows this relationship is subject-specific (39). This has led to application of subject-specific algorithmic estimation based on bed-side, *two-step calibration*, for immediate generation of tidal volume from EIT measurements. Similarly, estimation of heart rate from EIT measurements has been externally validated against traditional measurement methods (41).

Further work has focused on internal validation, centred on the repeatability of EIT measurements, and the robustness of these measurements in the face of changing conditions (position, ventilation, induced bronchoconstriction, age, species etc.) (10, 34, 42, 43). This demonstrates the value of EIT as a non-invasive monitoring tool of changing physiology. Extended exploration of different EIT variables, examining those that best explain the variance in the outcome measures, is being used to direct which variables generated by the EIT are most useful for monitoring in the live animal.

### Methodology Used in Data Analysis

Demonstration of the linear relationship of impedance and spirometric tidal volume has been verified by the application of simple linear regression. While the correlation coefficient is high, this would be expected since the two are actually measuring the same thing. So reliance on this as an assessment for assessing agreement between two methods is restricted.

However, linear regression does allow the linear relationship between impedance and spirometric tidal volume (VT) to be defined. The resultant equation can be used to allow estimation or extrapolation of VT from the impedance (a unit-less measurement). The VT calculated from the change in impedance (VT_EIT_) can then be compared to the VT determined by spirometry (VT_SPIRO_), allowing the two methods to be assessed for agreement. Exploration of this relationship over repeated studies, failed to discern a consistent relationship between different individuals and thus a generic algorithm could not be developed. As this is subject-specific, and likely condition specific, conversion of impedance change measured using EIT to VT requires *real-time* determination of the linear relationship.

Generation of a linear equation to explain the relationship is best performed on dense data that covers a range of measurements. Data points should not be falsely inflated by including replicates of the same measurement (these should be averaged and the average value plotted).

For validation of the use of EIT to estimate VT, comparison was made to VT_SPIRO_ measurements, and similarly for heart rate, using agreement analysis. Correlation analysis is not suitable for comparing measurements since it violates the fundamental assumptions of correlation (that the measurements are not co-linear) and it does not demonstrate agreement.

Bland-Altman analysis offers a visual, but semi-quantitative method for assessment of agreement that has become well-accepted in medical research.

## EIT Variables

### Global

As previously mentioned (see Section Single-Plane and Two-Plane EIT), EIT data can be collected using either single-plane or two-plane modalities. The data can then be analysed using the main ventilation signal (V), the cardiac-related signal in the lung ROI (Q), or the cardiac-related signal in the heart ROI (C) on a global scale. Some of the global variables described so far include tidal variation or volume, centre of ventilation, global inhomogeneity index, heart and respiratory rate (Beazley et al. In press) (30, 32).

### Regional

Once the data has been analysed globally, components of the signals can be analysed separately. For instance, regions describe the separation of the ventilation signal into typically eight distinct areas: the left (L) and right (R) lung region which is then subdivided into ventral (V), central ventral (CV), central dorsal (CD) and dorsal (D) regions. If two-plane EIT is used, the slice [cranial (Cr), middle (M) and caudal (Ca)] in the transverse plane can also be described.

### Pixels

These regions can be further divided into individual matrix lines based on all the individual pixels on a single horizontal or vertical line (28), or to the smallest level of individual pixels. Individual pixels represent an arbitrary grid of 32 × 32 pixels across the entire image. Output from the analysis of individual pixels may lead to pixel mapping or histograms representing individual pixel activity. Other regions may be defined by the variables themselves. For example, areas of the lung with similar impedance change can be grouped based on a percentage of maximum impedance change (34, 44) and this is used to evaluate stretch.

**Figure 10:**
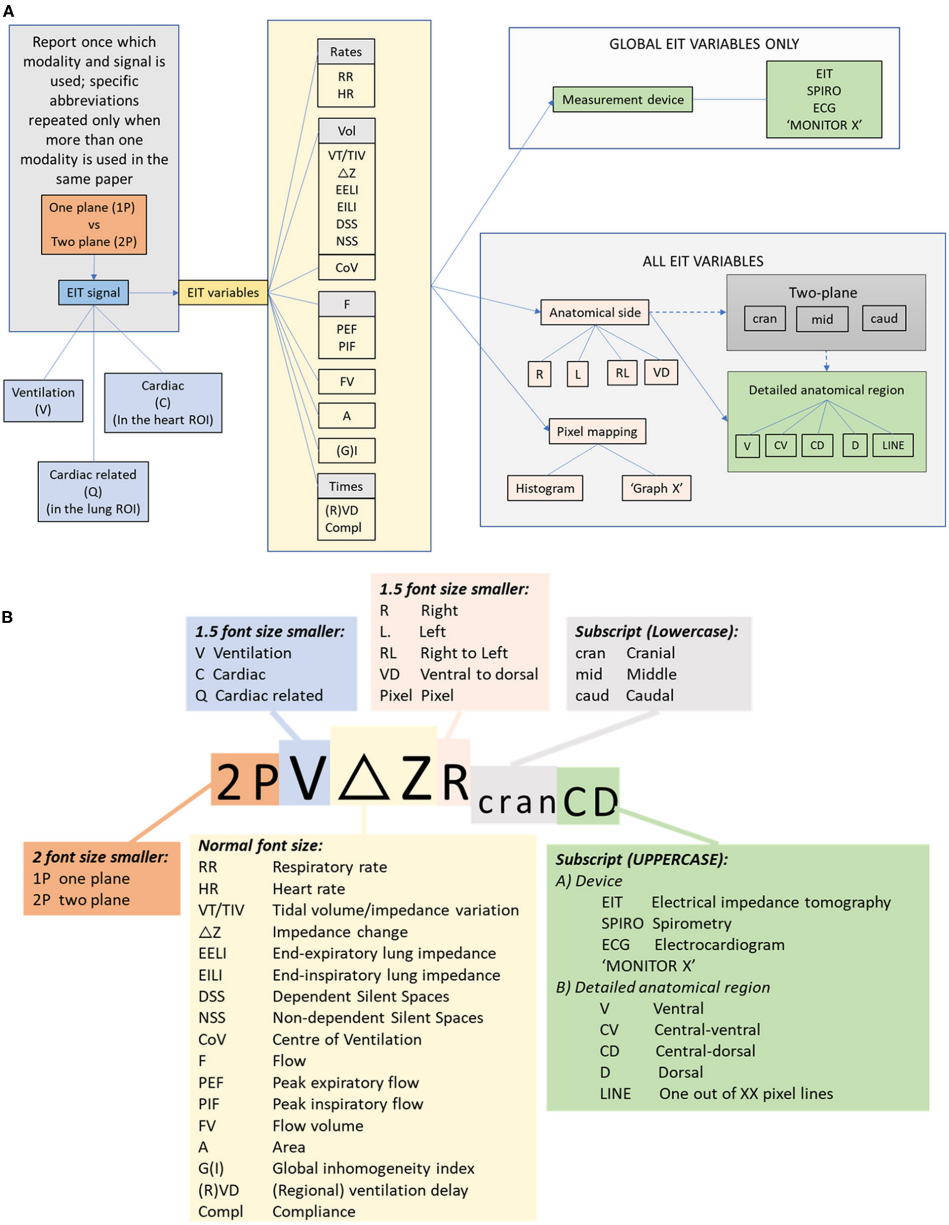
**(A)** Flow chart for description of EIT variables incorporating modality, signal, variable and localising descriptors. **(B)** Algorithm for abbreviation generation.

If EIT variables are compared with other technologies (for example spirometry), the variable should have the appropriate abbreviation for that technology included in each use of the terms. Figure 10 describes the nomenclature using a flow diagram. This algorithm can be followed when describing variables to ensure standardised reporting. EIT variables reported in the published literature thus far in veterinary medicine are described below.

### Functional Variables Described

#### Rates

Both heart and respiratory rates can be calculated using information from the lung and heart ROIs, respectively. Specifically, the respiratory rate is calculated from the number of end inspirations per minute. The heart rate can be evaluated using a combination of frequency filtering (where low frequency respiration is excluded) and selection of pixels from only the heart ROI. This region filtering is recommended as the amount of impedance change associated with cardiac activity is typically an order of magnitude smaller than that associated with respiration (2).

Respiratory rate measurement has been described in horses (10, 22, 43) and dogs (45). Heart rate measurement has been validated in horses against pulse rate derived from arterial blood pressure waveform recorded simultaneously (41).

#### Region


$$\begin{array}{l} (\text{ventral 25}\%\text{ = V};\text{ central ventral 25}\%\text{ = CV};\\ \text{ central dorsal 25}\%\text{ = CD};\text{ dorsal 25}\%\text{ = D}) \end{array}$$

The term ROI is used to describe the contour of anatomical structures as they pertain to FE models (meshes). The use of ROI to describe functional regional variables should be avoided, as it leads to confusing duplication. The term region should be used to describe a part of the functional image within the relevant ROI. The term ROI should only be used to describe the FE model (e.g., heart or lung ROI).

Regions should be divided as follows: To describe the ventro-dorsal distribution of ventilation in more detail the entire lung ROI is divided by three horizontal lines into four stacked portions of equal height. In the resulting eight regions, clear delineation between zones of differing ventilation is possible, while being sufficiently concise for presentation and comparison purposes. Defining left and right is necessary as ventilation of the right lung in most species is greater than in the left lung (22, 25, 34, 40, 46).

It is recommended to refer to regional ventilation in either the R or the L lung as follows: ΔZ(*R or L*)_*V*_, ΔZ (*R or L*)_*CV*_, ΔZ(*R or L*)_*CD*_, ΔZ (*R or L*)_*D*_.

Regions have been described in dogs undergoing recruitment manoeuvres (30), anaesthetised ponies in right lateral recumbency (10, 39, 47, 48), pregnant ponies (10, 18), horses post-anaesthesia (35), horses with cardiac disease (28), horses undergoing experimental bronchoconstriction (46) and anaesthetised rhinoceroses (21).

#### Variables of Linear-Plane Distribution

Occasionally, variables can be described using lines of pixels (i.e., a line of 1 x 32 pixels). Thus far, three methods of describing ventilatory impedance changes in a linear distribution have been reported:

##### Number of Ventilated Lines

All 32 horizontal matrix lines are assessed for each breath. The number of lines with detected impedance change is recorded for the right (TIV*R*_Line_) and the left (TIV*L*_Line_) lung field separately.

##### Average Maximum Impedance Change

The maximum impedance change is identified for each of the horizontal matrix lines (described above). The average of these maximums is calculated for each lung (right: Average maximum TIV*R*_Line_ and TIV*L*_Line_).

##### Graphical Illustration of Impedance Changes Over 32 Matrix Lines

The sum of impedance on a given line is plotted on a single axis, with each line on the opposite axis.

Examples of each of these variables has been used to describe detailed regional ventilatory changes in horses with cardiac disease (28).

### Ventilation

All of the following sections describe ventilatory variables. It is recommended that when non ventilatory variables are also reported (e.g., cardiac-related in the cardiac ROI or cardiac-related in the lung ROI), then V should precede acronyms specific to ventilation. Otherwise, the paper should report that all variables are derived from the ventilation signal once in the materials and methods section.

#### Tidal Impedance Variation or Tidal Volume (TIV or VT_EIT_, Respectively)

The total impedance change of all pixels between the beginning and end of inspiration within the lung ROI is calculated for each breath and can be used as a surrogate for tidal volume. This is calculated by subtracting the impedance (Z) at the beginning of inspiration from the impedance at the end of inspiration (also referred to as ΔZ). The term TIV should be used when the change in impedance is given in arbitrary units (AU).

Impedance change has been shown to have a direct relationship with tidal volume (VT) measured by spirometry in cattle, pigs, dogs and horses (39, 43, 49–51). However, there are inconsistencies in the literature in the terms used to describe this variable. The term “tidal volume” has been reported in dogs (40, 52) and horses (22, 35, 43, 46, 53), although volume was not actually directly measured. Delta Z has also been described without specifically being called tidal volume or variation (28, 45).

It is recommended that the term tidal variation (TIV) is used when the change in impedance is presented in AU, while the term tidal volume (VT_EIT_) is used when the change in impedance is converted to units of volume (e.g., L or mL), either using a predefined linear regression line or after calibration using spirometry (49). It should be specified in materials and methods if tidal variation (TIV in AU or tidal volume (VT_EIT_) in L or mL estimated from measured ΔZ) is used to estimate gas volume per breath. Direct display of VT_EIT_ by the EIT software on breath-by-breath basis in mL after an indirect two-point calibration has been described in pigs (54).

#### End-Expiratory Lung Impedance and End-Inspiratory Lung Impedance (EELI and EILI, Respectively)

The impedance at the end of each inspiration (EILI) or expiration (EELI) approximately represents the lung volume at that time of the respiratory cycle. As these variables are given as AU of impedance, the value of EELI and EILI lies in examining their trends over time, and their responses to certain interventions. Most commonly, EELI has been used to detect improvements in lung aeration (functional residual capacity; FRC) secondary to recruitment manoeuvres or changes in positive end-expiratory pressures (22, 40, 52). In addition, the change in EELI after injection of a contrast medium (typically an electrolyte-rich fluid like hypertonic saline) can be used to calculate changes in the cardiac-related signal in the lung ROI (21).

#### Dependent or Non-dependent Silent Space (DSS and NSS)

When areas within the lung ROI have impedance changes <10% of the maximum measured value, these areas are assumed to be areas of hypoventilation. The term Silent Spaces is used to describe these areas (44). When the areas are dependent (DSS; defined as below the ventilation horizon), the cause of low ventilation is often atelectasis (21), or intra-airway fluid accumulation (55). When the areas are non-dependent (NSS; i.e., above the ventilation horizon), over-distension may play a role (56).

Silent Spaces have been used to assess ventilatory changes in horses with heart disease (28), horses undergoing surgery (49) or anaesthetised horses undergoing mechanical ventilation with or without ARM under anaesthesia (38, 57), dogs undergoing PEEP challenges (32, 40), anaesthetised rhinoceroses (21) and an anaesthetised orangutan (25).

#### Centre of Ventilation [Ventral to Dorsal (CoV_VD_) and Right to Left (CoV_RL_)]

The ventral to dorsal (CoV_VD_) and right to left (CoV_RL_) centres of ventilation express the geometric focal point of overall ventilation as a single percentage. For CoV_VD_ and CoV_RL_ 0% refers to ventilation occurring in the most ventral region of the lung and the outermost region of the right lung, respectively, whereas 100% refers to that in the most dorsal region and the outermost region of the left lung, respectively (2, 58). This represents the focal point of the maximum impedance change and stands out as a relatively stable EIT variable. The centre of ventilation may remain largely unchanged, while other variables show significant changes in ventilatory distribution [for examples see (28, 46, 53)]. The line that runs horizontally through the CoVVD is known as the ventilation horizon.

**Figure 11:**
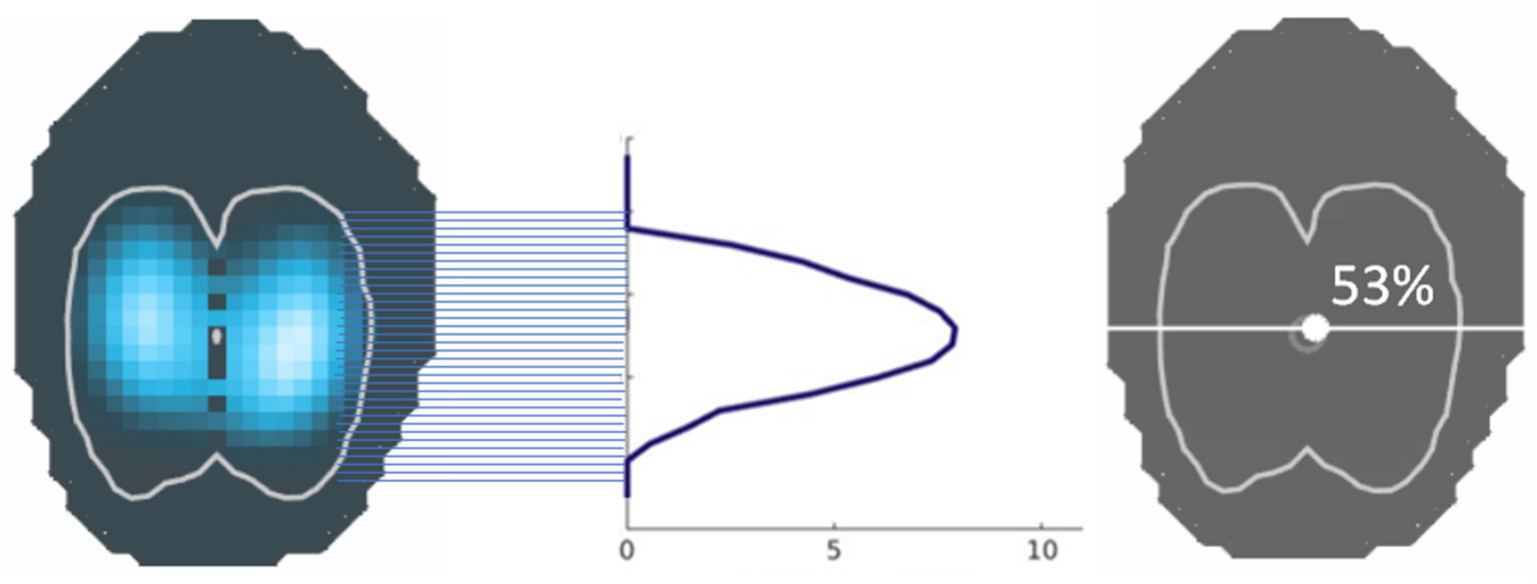
The centre of ventilation is calculated from the sum of impedance on a given pixel line, divided by the sum of all impedance, and given as a percentage. This can be performed in both the horizontal and vertical axes.

CoV – the image is divided into 32 horizontal (R to L) or 32 vertical (V to D) pixel lines. In each horizontal region, the image sum is determined and the CoV calculated using the formula


$$\begin{array}{l} \text{CoV\%}=\left(\text{Heightweightedpixelsum}\right)/\left(\text{Pixelsum}\right)\text{x}100, \end{array}$$

where Pixel sum = sum of all ΔZ and Height weighted pixel sum = pixel line number x ΔZ (Figure 11).

Examples in the veterinary literature describing the use of CoV include its use in evaluating distribution of ventilation in standing sedated horses (22), horses with cardiac disease (28), horses undergoing experimental bronchoconstriction (46), horses with asthma before and after exercise (53), anaesthetised horses with mechanical ventilation with or without various ARM (22, 38, 57), horses undergoing various surgical procedures (49), anaesthetised dogs in various recumbencies (42), dogs undergoing ARM or increasing PEEP (32, 40) and anaesthetised rhinoceroses (21).

#### Flow (F, PIF, PEF)

**Figure 12:**
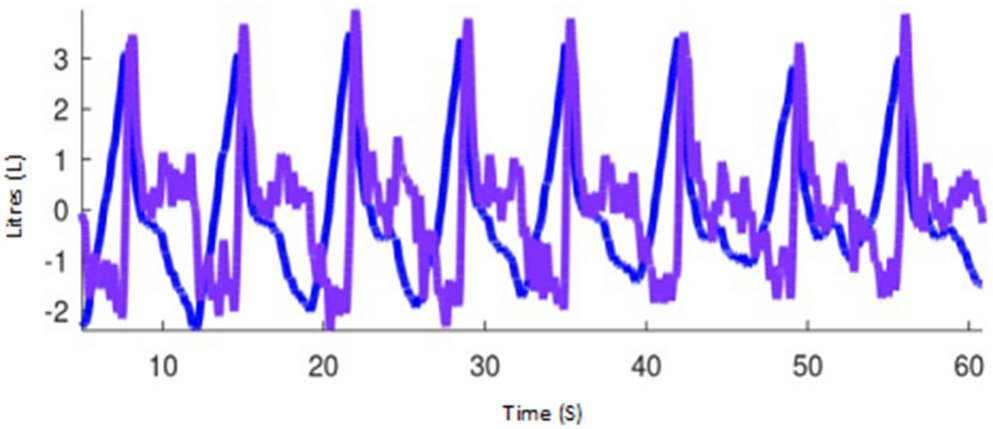
Impedance curves representing tidal impedance volume (the volume signal; blue) and the first flow derivative, flow (purple) using Octave software (GNU, public licence, USA).

Global peak inspiratory (PIF) and expiratory (PEF) gas flows are calculated as the first derivative of the global impedance change (Figure 12). The flow variables for inspiratory and expiratory flow have also been normalised to TIV (previously referred to as ΔZ) to account for differences in breath size between animals (PIF/TIV and PEF/TIV, respectively). Flow can be measured and reported as global (PEF/PIF), L or R lung region (PEF*R*/PIF*R* and PEF*L*/PIF*L*), and if available, vertical regions (i.e., PIF*R*_*CD*_), or pixel-level if appropriate.

Consistent with TIV, the acronyms PIF_EIT_ and PEF_EIT_ are recommended for when peak inspiratory and expiratory flows, respectively, are calculated as the first derivative of the calibrated global impedance change and expressed in units of flow (e.g., L/min), and compared to or calibrated with another device. When AU of impedance changes are used (i.e., without calibration), or when there is no other device compared, PIF and PEF should be used.

Flow can also be described for non-peak time points such as described by Secombe et al. (46), and should be abbreviated as F. The mean of the late phase of inspiratory and expiratory flow has been suggested as a fast method of evaluating flow changes in this phase of respiration (59). Other ventilatory flow variables described in veterinary medicine include the nadir point of biphasic expiratory flow (Byrne et al., in preparation).

Flow has been described using EIT in horses in a variety of clinical and experimental settings, as well as pigs (28, 43, 46, 53, 59).

#### Ventilated Area

The ventilated area is described by the percentage of pixels of the total image representing the ventilated area of the right (A_R_) and left (A_L_) lung field identified by detectable impedance change. Ventilated area is an example of a variable that does not require FE model application.

**Figure 13:**
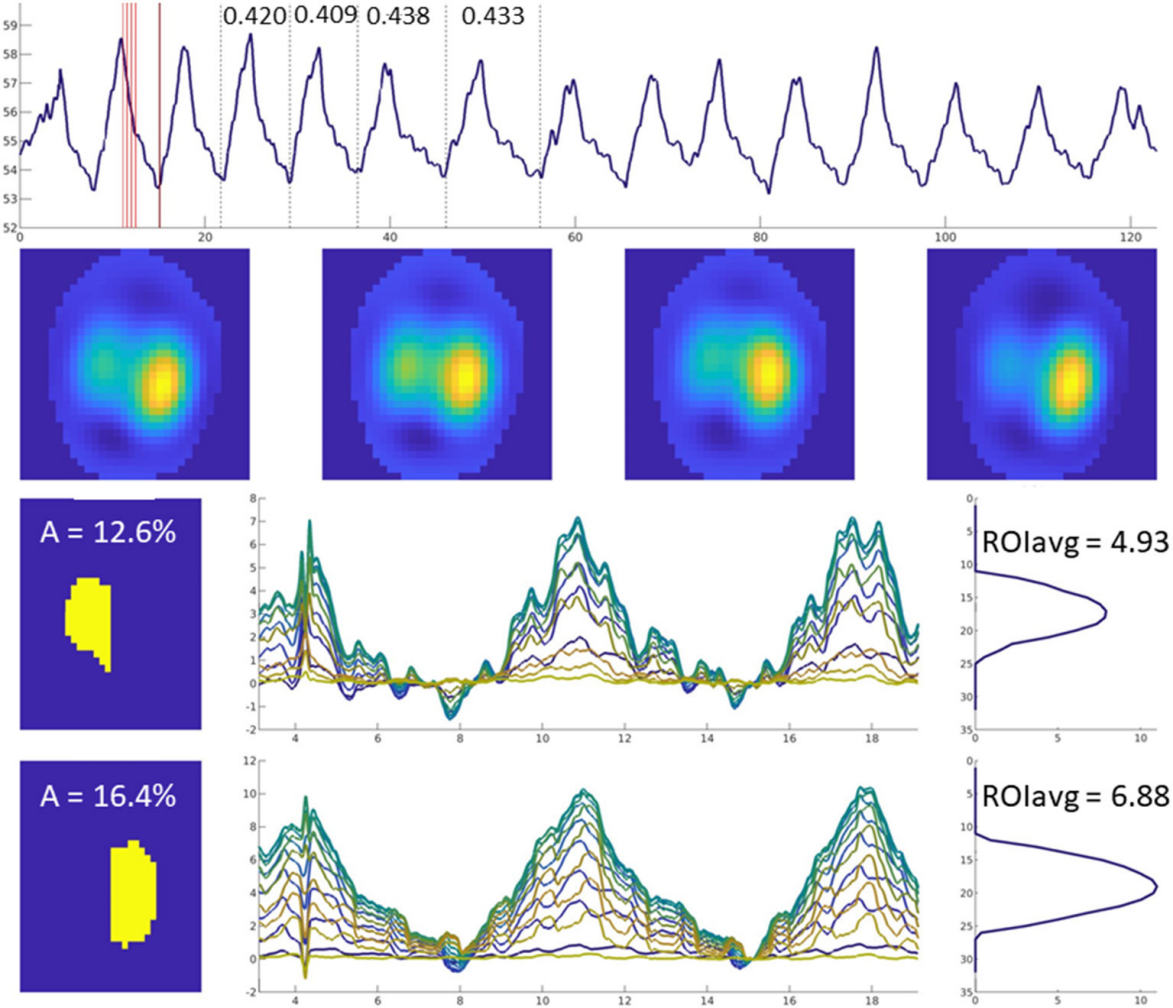
Images of four breaths are displayed in the upper horizontal row. The mean ventilated area is shown in the left-sided column in yellow (left lung top, right lung bottom image). Impedance change over time is graphically illustrated for each of the 32 lines in different colours.

Ventilated area has been used to describe ventilatory changes in horses with cardiac disease (28) (Figure 13) as well as in horses undergoing anaesthesia with or without mechanical ventilation (38).

#### Ventilated Area Ratios

The ratio of ventilation in the left to right lungs (A*R:L*), or from the dorsal half of both lungs to the ventral half of both lungs (A*V:D*) has been described. Left to right ratios have been described in standing awake horses at baseline, after sedation, and during rebreathing (22), in pregnant ponies (10), and anaesthetised rhinos (21). Dorsal to ventral ratios have been described in pregnant ponies (10) and preterm lambs (60).

#### Global Inhomogeneity Factor or Index (GI)

This variable describes the overall spatial heterogeneity of tidal volume distribution over a minimum of four breaths (61, 62). It is displayed as a single number whereby a higher value means greater inhomogeneity in aeration of lung units.

In veterinary medicine, GI has been described in anaesthetised dogs in various recumbencies (42), standing healthy horses (22) and horses with cardiac disease (28), as well as anaesthetised horses undergoing recruitment manoeuvres (22).

#### Flow – Volume Loop Variables (FV)

The flow volume loop can be calculated (FV) from the ventilation impedance signal and the volume-derived flow signal (Figure 12). Steepness of the slope of the initial expiratory part of the FV loop is described as the FV_slope_. The value of the steepest expiratory slope of all the flow-volume loops generated within the lung ROI's per breath is called the maximum FV_slope_. The intercept between the steep and the horizontal part of the expiratory FV loop (FV_intercept)_ describes the change between high-flow expiration and low-flow expiration. The relationship between the high- and low-flow expiration periods can be expressed as the ratio between intercept and the total tidal variation (FV_intercept_ / TIF; previously referred to as ΔZ).

Flow-volume loop variables have been used to describe change observed in horses with experimental histamine-induced bronchoconstriction and salbutamol-induced bronchodilation (43).

#### Regional Ventilation Delays (RVD)

**Figure 14:**
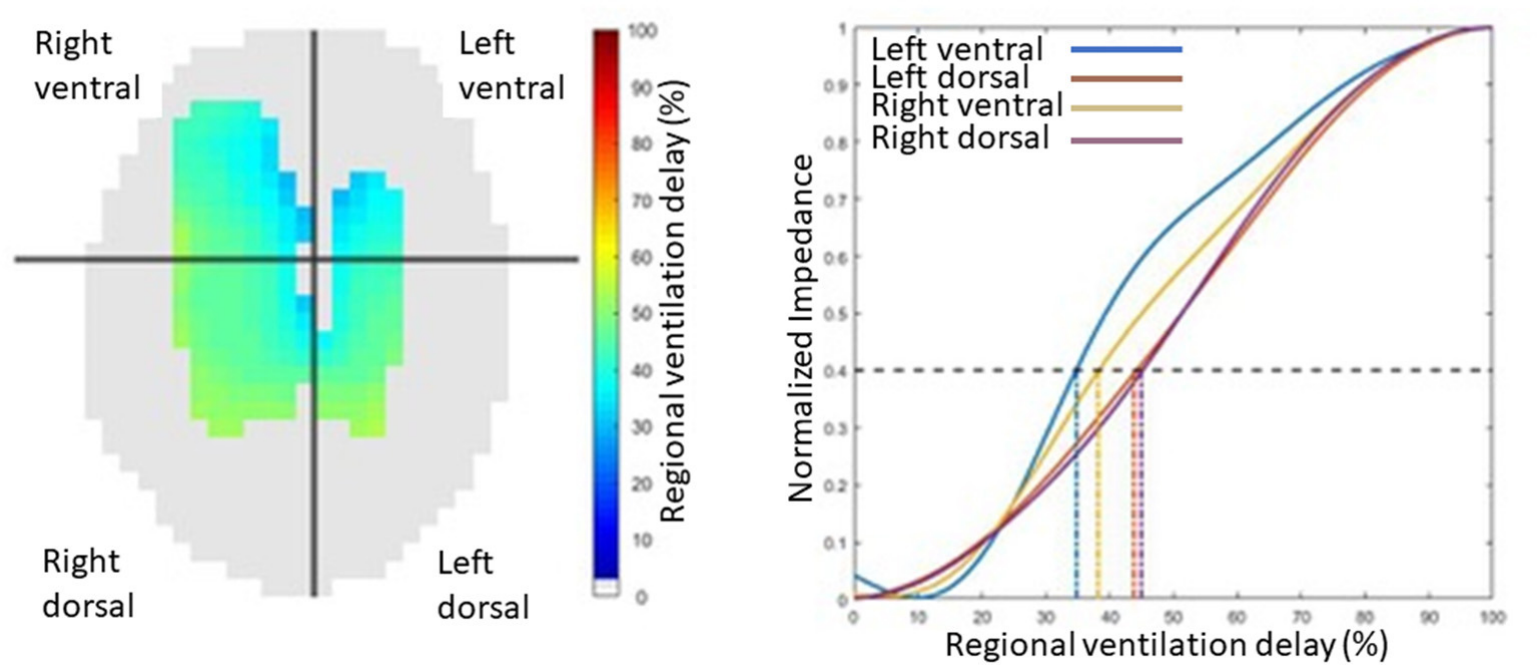
An illustration of regional ventilation delay showing the four regions, right ventral, left ventral, right dorsal and left dorsal regions, in pixel form on the left image and graphical form on the right.

While the global inhomogeneity index or factor describes the overall spatial inhomogeneity of lung unit ventilation, temporal inhomogeneity is described by regional ventilation delays. The RVD is the time for any regional impedance-time curve to reach a certain threshold of its maximal impedance change, as shown in Figure 14 (63). Regional time delays can be further indexed against the standard deviation of RVD in all pixels, with a small index indicative of relatively homogeneous regional lung unit filling and a large index indicating relatively inhomogeneous regional lung unit filling. This indexation allows for determination of whether there is cyclic opening/closing phenomena in very heterogeneous lungs.

Specific variables of regional time delays include those used to describe auto-recruitment in horses post-anaesthesia (35):

- Regional filling time was described as the time at which the total regional impedance change reached 50% of its maximum impedance change during inspiration normalised against the global inspiratory time.
- Regional inflation period was defined as the period during which the total regional impedance change remained above 50% of its maximum inspiratory impedance change, normalised against the global breath length.

Regional time delay and its inhomogeneity index has also been described in pigs in a model of lung injury during ARM (64).

#### Regional Expiratory Time Constants

Expiratory time constants in EIT typically refer to the heterogeneity of lung emptying. The volume expired in a given time can be calculated on a breath-by-breath basis. Time constant patterns can also separate lung diseases based on their underlying pathologies (i.e., restrictive vs. obstructive diseases) (65).

#### Regional Compliance

Global compliance can be calculated using EIT and simultaneously measured airway pressures. Pixel-level compliance has been calculated by dividing the total impedance change for that pixel by the airway pressure (32).

#### Relative Tidal Stretch (Stretch)

Stretch describes the change in impedance (presumed to be a surrogate for mechanical tissue expansion during inspiration) in the lung ROI during inspiration, expressed as a percentage of the maximum impedance change (34, 44). Stretch is displayed graphically both imposed on the lung ROI, as well as in bar chart form as deciles (i.e., the percentage of pixels that have an impedance change 30–40% of the maximum for that breath, for instance).

### Cardiac – Related Signal in Lung ROI

**Figure 15:**
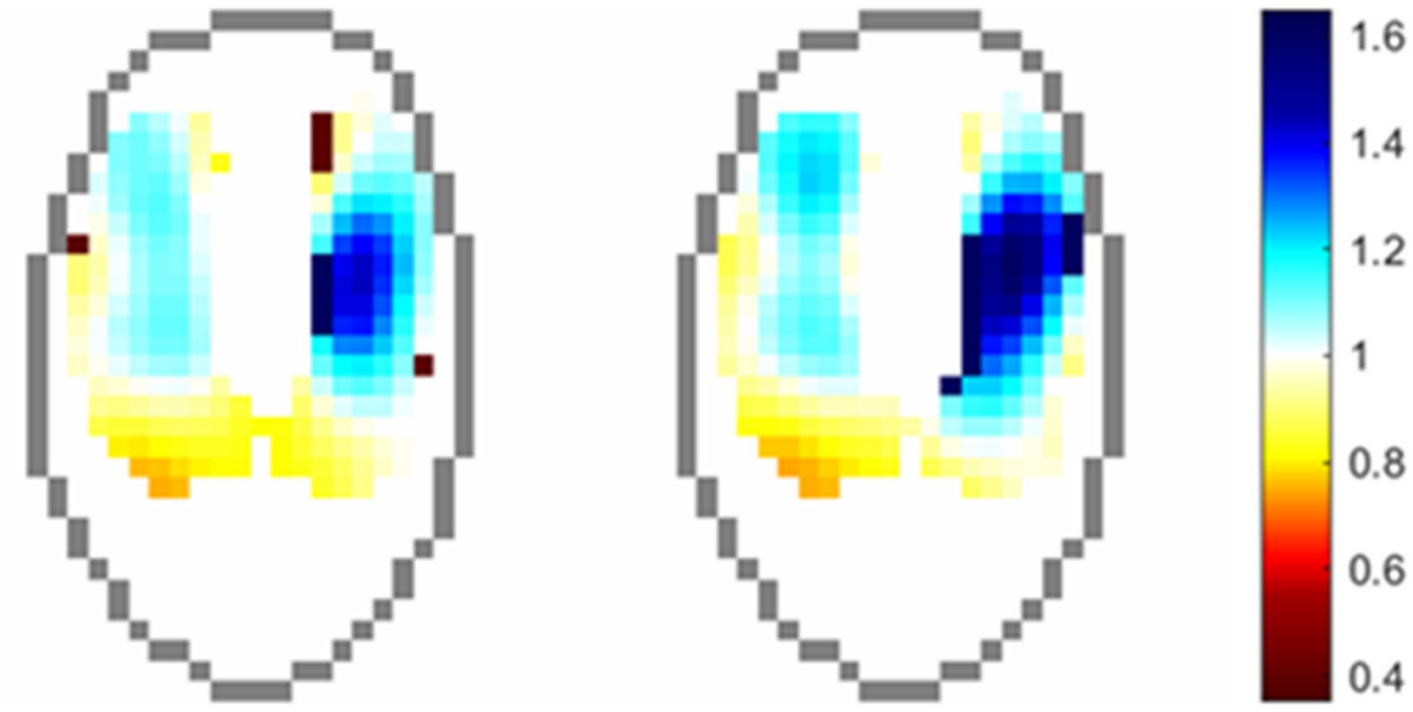
Regional distribution of VQ ratios in an anesthetised horse. Pixels values are the ratio of normalised ventilation to normalised perfusion at each time point.

Indicator solutions (typically high-concentrate salt solutions) increase the conductivity within the chest, leading to lowered electrical impedance, and thus correspond with lung perfusion as the solution traverses from the right heart into the pulmonary circulation. Frequency-filtering of data to remove the ventilation signal has also been shown to produce information relating to this signal, without the need for contrast solutions (66, 67). Evaluation of the cardiac-related signal in the lung ROI allows graphical representation of the ventilation perfusion (V/Q) mismatch as seen in Figure 15.

Evaluation of the change in impedance in the lung ROI after infusion of 23% hypertonic saline has been described in anaesthetised white rhinoceroses (21). In this paper, the cardiac-related signal in the lung ROI (described as perfusion) was evaluated by subtracting the maximal drop in global impedance from the reference baseline values.

Other papers in animals describing the cardiac-related signal in the lung ROI have mostly come from translational medicine papers (12, 67–72).

## Clinical Summary

The integration of thoracic EIT in veterinary diagnostics has shown promise as a clinically useful monitoring tool. The portable, non-invasive nature of EIT opens a broader range of applications available in veterinary medicine. To date, the majority of research has focused on validation of EIT as a clinically useful monitoring tool (31, 39, 48, 73) and the evaluation of ventilation distribution in a variety of settings (10, 21, 22, 38), with more recent developments in cardiac studies (41) and pulmonary pathology (3, 28, 43).

Thoracic EIT so far has been studied in a multitude of species (Appendix 2). There is scope for cross species benefits for ventilatory monitoring, the limitation being anatomical differences. To some extent this can be overcome using species-specific FE models and varying sizes of EIT belts. Other anatomical differences such as feathers and hair can be overcome by species-specific electrode design.

An additional challenge facing thoracic EIT in veterinary medicine is the environment in which data is gathered. In field settings, battery power has been utilised for measurements in cattle and foals, alleviating the necessity for mains power. This increases the breadth of locations where EIT can be used.

The future of veterinary thoracic EIT is exciting; improvements in data collection with refined belts and algorithm development will improve clinical usage. It is hoped that the two-plane EIT technology can revolutionise thoracic monitoring to provide true 3-D imaging allowing the whole lung field to be examined. Veterinary specific, user-friendly interfaces providing a wireless animal-side tool will aid short-and long-term thoracic and cardiac monitoring, with the additional application of lung pathology identification.

## Conclusion

While veterinary EIT is in its infancy, there are opportunities for valuable research and development to aid monitoring of ventilation in critical patients, more routine monitoring under anaesthesia and pathology identification. As more research is produced, there is a need to standardise nomenclature and recommend procedures. This consensus group hopes to have identified areas where terminology can be standardised and to have provided a summary of current practices and developments in veterinary thoracic EIT.

## Author Contributions

OB, DB, and MM were responsible for the collection of the responses and drafting of the manuscript. GH was responsible for the statistics section. OB, DB, MM, MS, JA, AAd, AAm, and FM contributed figures to the manuscript. OB, DB, MS, FM, JA, AW, JSc, GH, CB, UA, UB, NH, CS, AS, JSo, SB, CM, AAd, AAm, and MM were responsible for the review of the manuscript and preparation of the final manuscript. All authors contributed to the article and approved the submitted version.

## Funding

An exemption for the publication fees has been discussed by MM as part of the special issue for Veterinary Electrical Impedance Tomography.

## Conflict of Interest

The authors declare that the research was conducted in the absence of any commercial or financial relationships that could be construed as a potential conflict of interest. The reviewer ZZ declared a past collaboration with one of the authors AAd to the handling Editor.

## Publisher's Note

All claims expressed in this article are solely those of the authors and do not necessarily represent those of their affiliated organizations, or those of the publisher, the editors and the reviewers. Any product that may be evaluated in this article, or claim that may be made by its manufacturer, is not guaranteed or endorsed by the publisher.
