# Supplementary material for: Thoracic Electrical Impedance Tomography—The 2022 Veterinary Consensus Statement
Source: Front Vet Sci. 2022 Jul 22;9:946911. doi: 10.3389/fvets.2022.946911 (PMC9354895; doi:10.3389/fvets.2022.946911)
Supplement: Supplementary file 1 [file Data_Sheet_1.pdf]

| Reference | Year | Title | Animals | Experiment type | Setting | Primary Intervention | EIT system | EIT signal(s) | Significant EIT variables | Findings | Normal vs pathologic results |
|---|---|---|---|---|---|---|---|---|---|---|---|
| Schramel et al | 2012 | Distribution of ventilation in pregnant Shetland ponies measured by Electrical Impedance Tomography. | 6 healthy pregnant Shetland ponies | Experimental; Observational longitudinal | Conscious, standing | N/A | 32 electrodes, Dixtal Enlight | Ventilation | L:R, V:D, $\Delta Z_{region}$ | Ventilation reduced in the ventral lung fields as the time to parturition reduced | N/A |
| Tingay, Bhatia et al | 2014 | Effect of sustained inflation vs. stepwise PEEP strategy at birth on gas exchange and lung mechanics in preterm lambs | 19 preterm lambs | Translational; interventional trial | Anaesthetised? Sternal recumbency, intubated, mechanically ventilated | Stepwise PEEP increases or set PEEP | 16 electrodes, Goe-MF II | Ventilation | EELI; regional compliance | Greater non-dependent EELI in stepwise PEEP lambs | N/A |
| Tingay, Wallace et al | 2014 | Surfactant before the first inflation at birth improves spatial distribution of ventilation and reduces lung injury in preterm lambs | 18 preterm lambs | Translational; interventional trial | Anaesthetised, sternal recumbency, intubated, mechanically ventilated | Surfactant and sustained inflation | 16 electrodes, Goe-MF II | Ventilation | EELI; 32 line matrices for left and right lungs | L:R more homogenous in group which received surfactant prior to inflation | N/A |
| Moens et al | 2014 | Variety of non-invasive continuous monitoring methodologies including electrical impedance tomography provides novel insights into the physiology of lung collapse and recruitment–case report of an anaesthetized horse | 1 healthy adult horse | Experimental; interventional trial | Anaesthetised, right lateral recumbency, intubated, ventilated | Stepwise ARM and derecruitment maneouvres | 32 electrodes, Dixtal Enlight | Ventilation | dependent:non-dependent lung ΔZ; calculated 'compliance' | Delta Z increased in both lungs with stepwise ARM, and reduced with derecruitment. The dependent:non-dependent reduced with ARM. EIT 'compliance' reduced with ARM | N/A |
| Bodenstein et al | 2014 | Determination of respiratory gas flow by electrical impedance tomography in an animal model of mechanical ventilation | 13 healthy pigs | Translational; interventional trial | Anaesthetised, dorsal recumbency, intubated, mechaically ventilated | 6 had induced lung injury | 16 electrodes, Goe-MF II | Ventilation | Three vertically stacked lung regions, peak and late inspiratory and expiratory flows | Good to very correlation with spirometric flow; regional gas flow was distributed heterogeneously in different respiratory phases | Moderate lung injury led to reduced ventilation in the dependent lung region |
| Tingay et al | 2015 | An individualized approach to sustained inflation duration at birth improves outcomes in newborn preterm lambs | 24 preterm lambs | Translational; interventional trial | Anaesthetised, sternal recumbency, intubated, mechanically ventilated | Sustained inflation vs gradual tidal inflations | 16 electrodes, Goe-MF II | Ventilation | EELI, CoV, 32 line matrices for left and right lungs | Incremental increases in tidal volume led to more heterogeneity in EELI distribution | N/A |
| Nguyen et al | 2015 | Perfusion redistribution after a pulmonary-embolism-like event with contrast enhanced eit. | 8 adult sheep | Translational; interventional trial | Anaesthetised, intubated, mechanically ventilated, dorsal recumbency? | Right pulmonary artery embolage | 16-electrode KHU Mark 2.5 OR 32-electrode Swisstom Pioneer | Cardiac-related in the lung ROI | Peak of contrast dilution curve, maximum rate of uptake, maximum rate of washout in left and right lung fROIs | Larger volumes of lower concentration and smaller volumes of higher concentration saline performed similarly as contrast agents, | Perfusion, L:R perfusion, maximum uptake and washout were significantly different between baseline and embolised states |
| Ambrisko et al | 2016 | Assessment of distribution of ventilation by electrical impedance tomography in standing horses | 9 adult horses | Experimental; interventional trial | Standing, conscious; 6 horses had subclinical EA | Rebreathing, sedation | 32 electrodes, Timpel DX1800 | Ventilation | TIV, GI | CoV decreased and VT increased with rebreathing, reduced L:R with sighs (i.e, right-shift in ventilation) | N/A |
| Mosing et al | 2016 | Horses Auto-Recruit Their Lungs by Inspiratory Breath Holding Following Recovery from General Anaesthesia | 6 adult horses | Experimental; observational | Post-anaesthesia, standing, conscious | N/A | 32 electrode, Swisstom Pioneer | Ventilation | TIV, time above 50% maximum inspiratory impedance change, regions, regional filling time, regional inflation period | Breath holding occurred for up to 5 hours post-anaesthetic recovery. Filling time shortened in mid and ventral lung regions. Prolonged inflation period in some regions. | N/A |
| Miedema et al | 2016 | First Real-Time Visualization of a Spontaneous Pneumothorax Developing in a Preterm Lamb Using Electrical Impedance Tomography | Single preterm lamb | Translational; observational | Anaesthetised, intubated, mechanically ventilated, spontaneous pneumothorax | N/A | 32 electrode, Swisstom Pioneer | Ventilation | EELI, V:D | Increased impedance in left ventral lung, decreased EELI in rest of left lung. Decreased V:D | N/A |
| Ambrosio et al | 2017 | Ventilation distribution assessed with electrical impedance tomography and the influence of tidal volume, recruitment and positive end-expiratory pressure in isoflurane-anesthetized dogs | 14 healthy adult bitches | Experimental, interventional | Anaesthetised, intubated, mechanically ventilated, dorsal recumbency, ovariohysterectomy | ARM and PEEP, high and low tidal volumes | 16 electrodes, Dräger medical EIT evaluation kit | Ventilation | CoV, ΔZregion | ARM and PEEP shifted CoV dorsally during high tidal volume ventilation | N/A |
| Mosing et al | 2017 | Regional distribution of ventilation in horses in dorsal recumbency during spontaneous and mechanical ventilation assessed by electrical impedance tomography: a case series. | 4 adult horses | Experimental, interventional | Anaesthetised, spontenous breathing then mechanical ventilation, dorsal recumbency | Controlled mechanical ventilation | 32 electrodes, modified Swisstom Pioneer | Ventilation | CoV, DSS, NSS, A | CoV decreased towards non-dependent lung, DSS increased, NSS decreaed, A decreased | N/A |
| Gloning et al | 2017 | Electrical impedance tomography for lung ventilation monitoring of the dog. | 15 dogs | Experimental, interventional | Anaesthetised, sternal recumbency, intubated, mechanically ventilated | Stepwise PEEP increases | Unknown | Ventilation | ΔZ, ΔZregion | EIT recordings were useable without clipping the hair. PEEP led to increased impedance. Dorsal regions were better ventilated. | N/A |
| Ambrisko et al | 2017 | Assessment of distribution of ventilation and regional lung compliance by electrical impedance tomography in anaesthetized horses undergoing alveolar recruitment manoeuvres | 5 adult horses | Experimental, interventional | Anaesthetised, dorsal recumbency, intubated, mechanically ventilated | Stepwise PIP and PEEP increases and decreases | 32 electrodes, Timpel DX1800 | Ventilation | CoV, GI | CoV shifted dorsally and to the left at high airway pressures. | N/A |

| Reference | Year | Title | Animals | Experiment type | Setting | Primary Intervention | EIT system | EIT signal(s) | Significant EIT variables | Findings | Normal vs pathologic results |
|---|---|---|---|---|---|---|---|---|---|---|---|
| Ambrisko et al | 2017 | Impact of four different recumbencies on the distribution of ventilation in conscious or anaesthetized spontaneously breathing beagle dogs: An electrical impedance tomography study | 9 healthy beagles | Experimental, interventional | Conscious in various recumbencies, then anaesthetised in various recumbencies | Recumbency change, anaesthesia | 32 electrodes, Timpel DX1800 | Ventilation | CoV, GI | Variables did not change during recumbencies when conscious. CoV shifted dorsally when moving from dorsal recumbency to left lateral recumbency when anaesthetised | N/A |
| Thurk et al | 2017 | Effects of individualized electrical impedance tomography and image reconstruction settings upon the assessment of regional ventilation distribution: Comparison to 4-dimensional computed tomography in a porcine model | 13 landrace piglets | Experimental, observational | Anaesthetised, intubated, mechanically ventilated | N/A | 32 electrodes, Swisstom BBVet | Ventilation | ΔZ, 32 line matrices for left and right lungs | In comparison to 4DCT, EIT appropriate agreement for ventilation distribution | N/A |
| Mosing et al | 2017 | Ventilatory incidents monitored by electrical impedance tomography in an anaesthetized orangutan (Pongo abelii). | 1 orangutan | Clinical | Anaesthetised, intubated, spontaneous breathing | N/A | 32 electrodes, Swisstom BBVet | Ventilation | ΔZ, CoV, DSS, NSS, Stretch | One lung intubation identified by ROI delta Z, CT-identified pneumonia confirmed by DSS and Stretch, BALF associated DSS and NSS identified | Monitoring of real-time pathology |
| Meira et al | 2018 | Comparison of three continuous positive airway pressure (CPAP) interfaces in healthy Beagle dogs during medetomidine propofol constant rate infusions. | 10 healthy beagles | Experimental, interventional | Anaesthetised, spontaneous breathing, dorsal recumbency | Custom facemask, conical facemask, helmet | 32 electrodes, Swisstom BBVet | Ventilation | EELI, ΔZ, TIV, CoV, DSS, NSS | All masks led to increased EELI. There were minor changes in CoV using the helmet | N/A |
| Mosing et al | 2018 | Monitoring of tidal ventilation by electrical impedance tomography in anaesthetised horses. | 8 healthy adult horses | Experimental, interventional, crossover | Anaesthetised, intubated, spontaneous breathing, dorsal recumbency | CPAP | 32 electrodes, Swisstom BBVet | Ventilation | CoV, NSS, DSS, regions, fROI | CoV shifted to dependent lung and DSS smaller in CPAP | N/A |
| Auer et al | 2019 | Monitoring changes in distribution of pulmonary ventilation by functional electrical impedance tomography in anaesthetized ponies | 8 adult ponies | Experimental, observational | Anaesthetised, intubated, mechanical ventilation, right lateral recumbency | N/A | 32 electrodes, Swisstom BBVet | Ventilation | CoV, V:D, ΔZregion | Ventilation was heterogeneously distributed and predominantly right-sided and non-dependent. This distribution was apparent within 30 minutes of anaesthesia and persisted for the duration of anaethesia (90 minutes) | N/A |
| Mosing et al | 2019 | Monitoring of tidal ventilation by electrical impedance tomography in anaesthetised horses | 6 adult horses | Experimental, interventional | Anaesthetised, dorsal recumbency, intubated, mechanically ventilated | Stepwise increases and decreases in tidal volume | 32 electrodes, Swisstom BBVet | Ventilation | TIV | Tidal volume estimated by EIT was significantly correlated with tidal volume calculated by spirometry | N/A |
| Tingay et al | 2019 | Aeration strategy at birth influences the physiological response to surfactant in preterm lambs | 71 steroid-exposed preterm lambs | Experimental, interventional | Anaesthetised, dorsal recumbency, intubated, mechanically ventilated | No ARM vs sustained lung inflation ARM vs stepwise PEEP ARM. All groups subsequently received surfactant | 32 electrode, Swisstom Pioneer | Ventilation | EELI, EILI/TIV, CoV, ventral and dorsal regions divided by equal tissue volume (extrapolated from EIT data) | EELI increased in all groups similarly over time. Surfactant aided in redistribution of ventilation from predominantly non-dependent lung to become more uniform | N/A |
| Secombe et al | 2020 | Evaluation of histamine provoked changes in airflow using electrical impedance tomography in horses. | 6 healthy horses | Experimental, interventional | Conscious, sedated, standing, wearing facemasks with spirometry | Increasing concentrations of inhaled histamine were nebulised | 32 electrodes, Swisstom BBVet | Ventilation | PIF, PEF, $F_{region}$ | Flow increased with histamine bronchoprovocation. Expiratory flow changes best described the increased flow | N/A |
| Pleyers et al | 2020 | Investigation of selected respiratory effects of (dex)medetomidine in healthy Beagles | 8 health beagles | Experimental, invterventional, crossover | Consicous, sedated, right lateral recumbency | Sedation with dexmedetomidine or medetomidine | 32 electrodes, Swisstom BBVet | Ventilation | Minute ΔZ | Minute ΔZ was unchanged although ΔZ increased after sedation. There was no difference between dexmedetomidine and medetomidine | N/A |
| Dubo et al | 2020 | Low Spontaneous Breathing Effort during Extracorporeal Membrane Oxygenation in a Porcine Model of Severe Acute Respiratory Distress Syndrome | 12 sows | Experimental, interventional | Anaesthetised, intubated, lung injury, ECMO circuit | Near-apnoeic ventilation strategy or spontaneous breathing | 32 electrodes, Swisstom BBVet | Ventilation | TIV, EELI | There as more dorsal regional ventilation in the spontaneously breathing animals | Lung injury led to decreased EELI in both groups, with more central-ventral ventilation distribution |
| Mosing et al | 2020 | What hinders pulmonary gas exchange and changes distribution of ventilation in immobilized white rhinoceroses (Ceratotherium simum) in lateral recumbency? | 6 rhinos | Experimental, observational | Anaesthetised, intubated, spontaneously breathing, right lateral recumbency, intravenous hypertonic saline as contrast agent | N/A | 32 electrodes, Swisstom BBVet | Ventilation | A, CoV, NSS, DSS, ΔZregion, QΔZ | Shift in ventilation towards non-dependent lung. Perfusion similarly shifted towards the non-dependent lung, centred around the hilus. Breathholding redistributed ventilated towards the dependent lung | N/A |
| Brabant et al | 2021 | Effects of PEEP on the relationship between tidal volume and total impedance change measured via electrical impedance tomography (EIT). | 12 castrated cattle | Experimental, interventional | Anaesthetised, dorsal recumbency, intubated, mechanically ventilated, concurrent spirometry | Stepwise PEEP and VT increases | 32 electrodes, Swisstom BBVet | Ventilation | TIV | VT measured by spirometry and EIT were linearly correlated across a range of PEEP and VT settings | N/A |

| Reference | Year | Title | Animals | Experiment type | Setting | Primary Intervention | EIT system | EIT signal(s) | Significant EIT variables | Findings | Normal vs pathologic results |
|---|---|---|---|---|---|---|---|---|---|---|---|
| Crivellari et al | 2021 | Use of Electrical Impedance Tomography (EIT) to Estimate Tidal Volume in Anaesthetized Horses Undergoing Elective Surgery | 17 healthy horses | Clinical experimental, observational | Anaesthetised for elective surgery, dorsal recumbency, intubated, mechanically ventilated, concurrent spirometry | N/A | 32 electrodes, Swisstom BBVet | Ventilation | TIV | There were intraindividual differences in the slope of the linear relationship between $VT_{SPIRO}$ and $VT_{EIT}$ | N/A |
| Raisis et al | 2021 | The use of electrical impedance tomography (EIT) to evaluate pulse rate in anaesthetised horses. | 7 healthy horses | Experimental, interventional | Anaesthetised, dorsal recumbency, intubated, mechanically ventilated | Periodic apnoea, induced hypertension, induced hypotension | 32 electrodes, Swisstom BBVet | Cardiac-related in the cardiac ROI | HR | EIT HR had excellent agreement with arterial catheter pressure waveform pulse rates across baseline, hypotensive and hypertensive conditions | N/A |
| Secombe et al | 2021 | Can bronchoconstriction and bronchodilatation in horses be detected using electrical impedance tomography? | 17 healthy adult horses | Experimental, interventional | Conscious, sedated, standing, wearing facemasks with spirometry | Nebulised histamine to induce bronchoconstriction, nebulised salbutamol to induce bronchodilation | 32 electrodes, Swisstom BBVet | Ventilation | PIF, PEF, FV slope, FV intercept | PIF, PEF and FV slope significantly increased and the FV intercept normalised to VT decreased during bronchoconstriction. These changes returned to baseline after bronchodilation. VT did not change with bronchoconstriction or bronchodilation | N/A |
| Sacks et al | 2021 | Electrical impedance tomography to measure lung ventilation distribution in healthy horses and horses with left-sided cardiac volume overload. | 8 healthy adult horses, 4 horses with compensated LCVO, 2 horses with decompensated LCVO | Clinical, observational | Conscious, standing, pre- and post-furosemide (subset) | N/A | 32 electrodes, Swisstom BBVet | Ventilation | TIV, CoV, NSS, DSS, A, linear-plane distribution variables (32 line indices, average maximum linear plane distribution), GI, PIF (including normalised to VT), PEF (including normalised to VT), ΔZregion | Linear plane distribution and area variables were able to detect differences in ventilation distribution between healthy horses and horses with LCVO | LCVO had less left ventilated area, more right ventilated area, indices of linear plane distribution |
| Herteman et al | 2021 | Exercise-induced airflow changes in horses with asthma measured by electrical impedance tomography. | 9 healthy adult horses, 9 horses with MEA, 5 horses with SEA | Clinical, observational | Conscious, standing, before and after exercise | Exercise | 32 electrodes, Swisstom BBVet | Ventilation | PEF, PIF | PIF and PEF increased after exercise in horses with EA. MEA and SEA changes were not different | EIT was able to detect differences in flow between healthy horses and horses with EA after exercise |
| Bleul et al | 2021 | Assessment of Postnatal Pulmonary Adaption in Bovine Neonates Using Electric Impedance Tomography (EIT). | 20 calves | Experimental, observational | Conscious, sternal recumbency | N/A | 32 electrodes, Swisstom BBVet | Ventilation | TIV, RR, CoV, NSS, DSS, Stretch, ΔZregion | CoV was predominantly dorsal and right in neonates. RR decreased and VT increased from birth to 3 weeks | N/A |
| | | | | | | | | | | | |
| | | | | | | | | | | | |

ΔZ: change in impedance; 4DCT: Four dimensional computed tomography; A: ventilated area; ARM: alveolar recruitment maneouvre; BALF: bronchoalveolar lavage fluid; CoV: centre of ventilation; EA: equine asthma; ECMO: extracorporeal membrane oxygenation; EELI: End-expiratory lung impedance; F: Flow; fROI: functional ROI; FV: flow volume; GI: inhomogeneity index; HR: heart or pulse rate; N/A: Not applicable; L:R: ratio of ventilation of the left to right lung ROIs; LCVO: left-sided cardiac volume overload; PEEP: Positive end-expiratory pressure; PEF: Peak expiratory flow; PIF: Peak inspiratory flow; PIP: Peak inspiratory pressure; QΔZ: the change in impedance due to the cardiac-related signal in the lung ROI; ROI: region of interest; TIV: tidal impedance variation; V:D ratio of ventilation of the ventral to dorsal half of the lung ROI; VT: tidal volume; $VT_{EIT}$: VT calculated by EIT; $VT_{SPIRO}$: VT calculated by spirometry
