# Supplementary material for: Thoracic Electrical Impedance Tomography—The 2022 Veterinary Consensus Statement
Source: Front Vet Sci. 2022 Jul 22;9:946911. doi: 10.3389/fvets.2022.946911 (PMC9354895; doi:10.3389/fvets.2022.946911)
Supplement: Supplementary file 2 [file Data_Sheet_2.pdf]

Appendix 2. Guide to general belt usage from consensus participants

General Belt Usage	
Species	The majority of the members of the consensus group have worked with horses (15/20) (A, B, C, D, F, H, I, J, L-T). Seven have worked with dogs (A, C, D, H, K, M, S), four have worked with cattle (G, L, M, S) five with pigs (C, H, M, N, T), two with sheep (M, N), two with lambs (A, T) two with calves (E, M) three with rhinoceros (M, O, T), three with foals (I, M, O), two with cats (B, S), one with chickens (M) and two with an orangutan (M, O).<br><br>**The consensus group recommends the use of EIT in a variety of species.**
In which settings has EIT been used?	Members of the group have used EIT in experimental (16/20) for clinical (11/20) and field studies (5/20). Members used EIT in conscious (14/20), anaesthetised (12/20) and sedated animals (11/20).<br><br>**The consensus group recommends use of EIT in a variety of settings.**
EIT device	Members of the group have used the Pioneer (7/20) or BBvet (11/20) (Sentec, Landquart, Switzerland). There are some with experience in other devices including, Enlight (Timpel, Sao Paulo, Brazil) (D,R), Goe MF II (CareFusion, Höchberg, Germany) (A,S) and PulmoVista® 500 (Dräger, Lübeck Germany) (A,B).
Belt Design	**Belt configuration:** all of the group have experience with the 32-electrode configuration. Two have experience with the Drager (A, B) two with the Goe MF II (A, T) belts which are a 16-electrode configuration. Few members have used 2-plane EIT, either two belts containing 16 electrodes on each, or a wider belt with 16 cranial and 16 caudal electrodes (A, C, G, I, M).<br><br>**Belt material:** belt material most commonly used is neoprene (11/20); other materials used include rubber

	(5/20) and stretchable elastic fabric (9/20), fabric belts provided by the manufacturer (Sentec, Switzerland) work well in cats and small dogs (1/20) (R).<br><br>**Electrode material:** gold-plated (7/20), brass (2/20), stainless steel (8/20), unknown metal (2/20), galvanized steel (2/20) has been used.<br><br>**Shape of electrode:** the majority use round flat washers (11/20) however alternative designs used include small pins (M, T), spikes (G), custom-made screws (D) and rectangular shape (J, P).<br><br>**Site of closure:** the information provided by the group suggested that site of closure was equally split between the ventral (sternum) (7/20) and dorsal (spinal process) (8/20); the site of closure was not specified for all belts used.<br><br>**The consensus group recommends that the site closure should be species-specific. For the correct belt positioning and image reconstruction Electrode 1 should be on the left-hand side of the sternum.**
