# Supplementary material for: Thoracic Electrical Impedance Tomography—The 2022 Veterinary Consensus Statement
Source: Front Vet Sci. 2022 Jul 22;9:946911. doi: 10.3389/fvets.2022.946911 (PMC9354895; doi:10.3389/fvets.2022.946911)
Supplement: Supplementary file 3 [file Data_Sheet_3.pdf]

Appendix 3. Guide to patient and belt preparation, positioning and commonly recorded variables from members of the consensus group

Specific Belt Preparation and Positioning	
Animal preparation before belt placement	Skin preparation used by members of the consensus group includes:<br><br>**Wetting the skin with water** (10/20)<br><br>**Applying gel:** (15/20) The gel used was either low conducting ultrasound gel or obstetric gel. Reasons for choice include that these gels are thicker in consistency, water soluble with low conducting properties (G, H, I, M, N, O).<br><br>**Clipping of the hair:** was performed by 9/20 individuals in a variety of species (dogs, pigs, calves, horses and lambs). **Hypertonic saline** was used by one member in horses (C).<br><br>A cleaning agent containing seven parts ethanol (isopropanol) 96% and three parts hypertonic saline can be used (Q).<br><br>**Normal saline** was used by another member in horses (A).<br><br>**Parting wool** in adult sheep allowed better skin contact (N).<br><br>Note: It was observed that when using the BBVet that using too much ECG conductive gel resulted in shorting of the electrodes although this device uses a "skip 4" electrode current (M).<br><br>**The consensus group recommends the most important preparation prior to belt placement is cleaning and wetting of the skin. In longer haired animals the addition of low conducting or obstetric gel to the belt is recommended to provide adequate skin contact and avoid air between the electrode and skin.**

Landmarks for positioning	Landmarks commonly used by the consensus group members include the intercostal spaces. The space ranged from the 5th intercostal space (ICS) (7/20) to the 6th ICS (6/20).<br><br>Other landmarks used in different species include:<br><br>**Horses:** the axillary space (C), 3 fingers cranially from the xyphoid process in ponies (D), 5-6th ICS and the level of the tuber ischium in foals (N), behind the point of the elbow as far cranially as possible (L), behind the olecranon and the withers (P), 3 fingers caudal to the elbow at the 6th ICS at half the thorax height in horses.<br><br>**Dogs:** sternum-length in cm x 0.17 = cm cranial to xyphoid and transversal to chest perpendicular to the spine (M).<br><br>**Chickens:** the most cranial point of the sternum, transverse to the thorax and perpendicular to the spine.<br><br>**Rhino:** 10cm caudal to the point of the elbow, transverse to the thorax and perpendicular to the spine (M).<br><br>**The consensus group recommends vertical placement of the belt in line with the 5-6th intercostal space at the midpoint of the lateral thorax in large animals and small ruminants, and as per above in dogs and chickens. No information for other species is available.**
Positioning of animal	The consensus group members have experience in all positions with EIT. The most used position is standing (15/20) followed by dorsal (11/20), sternal (4/20) and lateral (5/20) recumbencies.
What data is routinely recorded besides EIT data?	Common parameters recorded by members included:<br><br>**Signalment:** weight (7/20), height (3/20), circumference of thorax (6/20), body condition score (7/20), age (6/20) and gender (6/20).<br><br>**Physical Examination:** heart rate (11/20), respiratory rate (8/20), temperature (5/20), ocular and nasal discharge

	(1/20), lung auscultation (1/20) and Horse Owner Assessed Respiratory Signs Index (HOARSI) score (1/20).<br><br>**Reference Devices:** spirometry (13/20), volumetric capnography (6/20) blood pressure (7/20) and blood gas data (10/20).<br><br>**Additional Recorded Variables:** BAL cytology (2/20), tracheal mucus scores (2/20), flowmetric plethysmography (1/20), body/ head position (1/20), flow-volume curves (1/20), oesophageal pressure (2/20), ultrasound images of the lungs (1/20), cardiac output (1/20).
