# Supplementary material for: Thoracic Electrical Impedance Tomography—The 2022 Veterinary Consensus Statement
Source: Front Vet Sci. 2022 Jul 22;9:946911. doi: 10.3389/fvets.2022.946911 (PMC9354895; doi:10.3389/fvets.2022.946911)
Supplement: Supplementary file 4 [file Data_Sheet_4.pdf]

Appendix 4. A guide to tips and tricks for successful data capture from members of the consensus group

Tips and Tricks for Successful Recording	
Tips and tricks have been collated and attributed to the corresponding member of the group so that further information can be sought from the relevant member if necessary. They have been grouped into preparation before belt placement, belt placement and animal positioning.	
Preparation before belt placement	**Preparation before belt placement**<br><br>**Wetting or moistening of the hair or coat** is performed by the majority of the consensus group prior to belt placement; this will help to reduce skin contact impedance (C, E, G, I, J, L, M, N, O, P). Water is preferred (over low conducting ultrasound gel) when animals have thick winter coats or sensitive gold-plated washers are used on short coats (H, O, P).<br><br>**Low conducting/ ultrasound gel** is also used by numerous members to improve skin contact (B, E, F, G, H, I, J, M, O, T).<br><br>Note: shorting has been observed between electrodes when too much gel is placed (J, O).<br><br>Others have used cloth embedded in hypertonic saline (7.2%) to improve skin contact (C).<br><br>Clipping or clip marks prior to belt placement is used by three members to ensure the belt is placed correctly, especially if the animal is to be moved or the belt removed and replaced multiple times. It is not essential to clip for measurements, but some recommend it for an improvement in skin contact (H, I, M, O).<br><br>Other methods to improve skin contact include placing conforming bandage over the belt and is

	used by some members of the group in horses (C, F, M, O, R) and dogs in dorsal recumbency (K).<br><br>Note: the bandage should not restrict thoracic movements.<br><br>Belt preparation is also important, the recommendation by some members is to make sure the belt is dried between animals and to place gel on the belt rather than the animal with or without sponges around the electrodes (G, H, L, M). This has been applied to horses and cattle (G, H, L, M).<br><br>**The recommended preparation will vary with species and coat length.**
Belt placement	**Belt placement performed by members varies with awareness state of animal**<br><br>**Conscious animals:** some recommend an adjustment period after placement to get the animal used to the belt of up to 10 minutes prior to data recording (E). This has been previously done successfully in calves. Others recommend training the animals with the belt prior to the study (Q). In horses, foals, cattle, and calves the belt can be placed on conscious animals and is generally well tolerated (E, F, G, M, O).<br><br>**Sedation:** administration of sedative prior to belt placement is preferred in conscious in animals that are not calm. In sheep, pigs, dogs and rhino the belt is placed after heavy sedation or induction (H, K, M, N, O).<br><br>**Anesthetised animals:** the method used depends on species and temperament.<br><br>**Horses:** Most of the consensus group place the belt after sedation and prior to anaesthesia (A, B,

	H, L, M, O, Q, R). Some have placed the belt post induction (C, D, S) however difficulties arise when placing the belt under the body (A).<br><br>**Larger animals such as cattle or rhino**: the use of a metal rail can be employed to allow easier belt placement after induction of anaesthesia. The animal is positioned in lateral recumbency over the metal rail which has been placed on the ground at the 5th ICS. This allows safe, easy and efficient belt placement (M, O, T).<br><br>**Belt orientation:** the correct orientation of the belt is important. When placed it is recommended to determine that right and left are correct on the animal and the corresponding SD images (G, I, L, M, N, S, T); this is most often done by pulling the belt away from the animal and checking on which side the failing electrodes are displayed on the screen. This also applies to the specific adapter provided with the hardware, this must be placed the correct way up (R).<br><br>**Belt fastening:** the preferred position of the fastener varies between conscious and recumbent animals. The preferred method used for placement in conscious animals such as cattle when standing is to use a hook to reach for the belt under the animal and then fasten dorsally or laterally to make placement as safe as possible (G, M).
Belt location	To ensure consistent belt location when moving animals, clip marks are useful (G, L, M, N, O). Electrode skin contact may need to be verified again by visual assessment of the bedside monitor.

Animal positioning	**Protection:** when animals are in dorsal or lateral recumbency placement of a foam strip or vacuum mattress between the belt and the table provides some padding to prevent skin damage (G, K, M, T).<br><br>**Head position:** the head must be straight to prevent shifting of ventilation and non-representative data. (I, M, R).
