# Supplementary material for: Thoracic Electrical Impedance Tomography—The 2022 Veterinary Consensus Statement
Source: Front Vet Sci. 2022 Jul 22;9:946911. doi: 10.3389/fvets.2022.946911 (PMC9354895; doi:10.3389/fvets.2022.946911)
Supplement: Supplementary file 5 [file Data_Sheet_5.pdf]

Appendix 5. Guide to data analysis from members of the consensus group

Data Analysis	
What analysis software to use	Of the software available, the most commonly used is IBEX, (current version 2019) (Sentec, Switzerland) (18/20), with some use of Matlab (Mathworks®, USA) (5/20), Octave (GNU public licence USA) (3/20) and customised software through EIDORS (GNU public license, USA) (2/20), STEM (2/20) and Dixtal (1/20). An older software GEOMFII was also used. (1/20).<br><br>**The consensus group recommends that for standard data analysis IBEX is sufficient. However, other software may be needed for species not commonly studied and for complex analyses multiple software programs may need to be employed.**
How do you reconstruct the images?	All members of the consensus group use the GREIT reconstruction algorithms and some then use species-specific FE models to define lung contours (5/20).<br><br>In birds, a region of interest has been used to eliminate the lung, as gas flows in one direction expanding air sacs outside the desired region (M).<br><br>Some exclude the pixels derived from the heart signal (B, N) or the rumen (G, M, N).<br><br>For heart rate analysis, the pixels derived from the heart or perfusion signal are selected (M, N, T)<br><br>**The consensus group recommends the use of GREIT reconstruction algorithms be used with species-specific FE models, as appropriate for the variables in question. Further regions can be applied if specific spatial information is required.**

How many breaths do you analyse per measurement?	The range of breaths selected by the consensus group for analysis varies from a minimum of three to a maximum of 22 breaths, with 10 breaths most analysed (17/20). Ideally, selected breaths should be consecutive (17/20). The time taken for data collection varies from the duration of a single breath up to ten minutes. The most common duration of data recording is a minimum of two minutes of stable breathing (taken from anesthetised animals whose respiratory rates give 10 breaths for analysis over that time).<br><br>**The consensus group recommends a minimum of 5 viable breaths with 10 breaths ideal for analysis within three-five minutes of the same ventilatory and cardiovascular condition to allow exclusion of artefactual breaths. The recommendation for analysis is ideally consecutive breaths.**
How do you decide which breaths to analyse?	All members select breaths visually within IBEX software, with breaths selected being consistent, artefact-free and where there is no electrode or contact failure. 'Artefact-free' breaths are defined by the smooth shape of the global impedance curve.<br><br>Some select breaths based on the ratio of the end expiratory lung impedance (EELI) at the start and end of the breath (A, I). For the selected breath, the start of breath EELI should differ by no more than 1/3 of the total impedance change for that breath using the IBEX software (Fig 9) (A, I, L).<br><br>Another member suggested that once the breaths have been selected, the data is exported to a spreadsheet software program and any obviously erroneous breaths deleted (O).<br><br>**The consensus group recommends selecting breaths that are uniform in shape on visual inspection, representative, regular and artefact-free breaths. Breaths can be included / excluded based on the shape of the global impedance curve.**

Variables used in analysis	The consensus group members have had experience in all of the currently available conventional variables and also some more specific variables designed for specific animal studies. (Section 5)<br><br>**Currently, veterinary thoracic EIT has assessed ventilator signals, cardiac-related signals in the lung ROI, and cardiac-related signals in the cardiac ROI.**
