# Supplementary material for: Thoracic Electrical Impedance Tomography—The 2022 Veterinary Consensus Statement
Source: Front Vet Sci. 2022 Jul 22;9:946911. doi: 10.3389/fvets.2022.946911 (PMC9354895; doi:10.3389/fvets.2022.946911)
Supplement: Supplementary file 6 [file Data_Sheet_6.pdf]

Appendix 6. Key to authors for Appendix 1-4

Key to Authors	
A	A. Adler
B	A. Ambrosio
C	J. Araos
D	U. Auer
E	U. Bleul
F	S. Beazley
G	O. Brabant
H	C. Braun
I	D. Byrne
J	N. Herteman
K	C. Meira
L	F. Moreno Martinez
M	M. Mosing
N	A. Raisis
O	M. Sacks
P	A. Schoster
Q	J.P. Schramel
R	C. Secombe
S	J. Soares
T	A.D. Waldmann
